# Malaysian Expert Consensus Guidelines for the Diagnosis and Management of Multiple Myeloma: A Modified Delphi Approach

**DOI:** 10.21315/mjms-03-2026-153

**Published:** 2026-08-31

**Authors:** Gin Gin Gan, Lily Lee Lee Wong, Mohd Haris Fadzillah Abdul Rahman, Yang Liang Boo, Lee Ping Chew, Kim Wah Ho, Azlan Husin, Jay Suriar Rajasuriar, Sen Mui Tan, Sui Keat Tan, Tengku Ahmad Hidayat K Aziz Tengku

**Affiliations:** 1 Department of Medicine, Faculty of Medicine, Universiti Malaya, 50603 Kuala Lumpur, Wilayah Persekutuan Kuala Lumpur, Malaysia; 2 Haematology Unit, Queen Elizabeth Hospital, 88586 Kota Kinabalu, Sabah, Malaysia; 3 Haematology Unit, Subang Jaya Medical Centre, 47500 Subang Jaya, Selangor, Malaysia; 4 Haematology Unit, Department of Medicine, Sultanah Aminah Hospital, 80100 Johor Bahru, Johor, Malaysia; 5 Department of Medicine, Haematology Unit, Sarawak General Hospital, 93586 Kuching, Sarawak, Malaysia; 6 Haematology Unit, Sunway Medical Centre, Bandar Sunway, 47500 Subang Jaya, Selangor, Malaysia; 7 Department of Internal Medicine, School of Medical Sciences, Universiti Sains Malaysia, Health Campus, 16150 Kubang Kerian, Kelantan, Malaysia; 8 Haematology Unit, Gleneagles Hospital, 50450 Kuala Lumpur, Wilayah Persekutuan Kuala Lumpur, Malaysia; 9 Haematology Unit, Icon Oncology, Island Hospital, 10450 George Town, Pulau Pinang, Malaysia; 10 Haematology Unit, Beacon Hospital, 46050 Petaling Jaya, Selangor, Malaysia

**Keywords:** consensus, multiple myeloma, Malaysia, treatment, management, risk stratification

## Abstract

The rising incidence of multiple myeloma (MM) in Malaysia, combined with limited access to newer therapies, highlights the need for locally adapted clinical guidance. This consensus aimed at establishing expert recommendations for the management of MM within Malaysia’s resource-variable healthcare system. A modified Delphi process was conducted with 11 haematologists. To inform evidence-based recommendations, a targeted literature review was performed from January 2012 to June 2025. These recommendations were then evaluated using the Grading of Recommendations, Assessment, Development and Evaluation (GRADE) criteria. A total of 58 consensus statements were developed across various areas, including diagnosis, staging, treatment of newly diagnosed and relapsed/refractory disease, renal impairment, infection prevention, high-risk smouldering myeloma, and supportive care. Agreement was assessed using a three-point Likert scale, with all statements achieving ≥ 80% consensus. Consensus recommendations highlighted that triplet induction remains the minimum standard for transplant-eligible patients in Malaysia. Quadruplet regimens deliver deeper responses and improved survival but are limited by regulatory and cost barriers across Asian health systems. In transplant-ineligible patients, treatment should be individualised based on age, frailty, cytogenetic risk, comorbidities, and financial considerations. Novel cellular and immune-based therapies, including CAR-T cells and bispecific antibodies, offer meaningful benefit in heavily pretreated disease, though availability remains restricted. Infection prophylaxis, immunoglobulin support, vaccination, and proactive management of myeloma-related bone disease are essential components of comprehensive care. Continued efforts to improve access to advanced diagnostics, novel therapies, and clinical trial participation will be critical to further enhance outcomes. This consensus provides a standardised, evidence-based framework to optimise MM care and improve patient outcomes nationally.

## Introduction

Multiple myeloma (MM) is a malignant plasma cell disorder characterised by the uncontrolled proliferation of abnormal plasma cells (1). Globally, MM ranks 21st in incidence and 17th in cancer mortality among all cancers, with age-standardised rates of 1.8 and 1.1 per 100,000 population, respectively (2). Asia accounts for the largest share of cases, with 37.1% of all global 5-year cases worldwide (2–4). Males consistently show a higher incidence, mortality rate, and disability-adjusted life years (DALYs) compared to females (5). In Malaysia, the incidence of MM has risen from 0.98 to 1.04 per 100,000 population between 2005 and 2019, whereas prevalence rates rose from 2.05 to 2.39 per 100,000 population during the same period (6). According to the International Agency for Research on Cancer (IARC) GLOBOCAN 2022 statistics, the 5-year prevalence of MM in Malaysia was reported to be 1,010 cases (3.0 per 100,000). Worldwide, age-standardised prevalence and DALYs have all shown an upward trend from 1990 to 2021 (2–4).

The newer generation of therapies, such as proteasome inhibitors (PIs) (e.g., carfilzomib and ixazomib), immunomodulatory drugs (IMiDs) (e.g., lenalidomide and pomalidomide), and anti-CD38 monoclonal antibodies (mAbs) (e.g., daratumumab), has transformed the treatment landscape of MM. Patients today have an improved chance of achieving remission and prolonged survival (7, 8). However, many of these novel therapies remain inaccessible in low- and middle-income countries because of their high cost (8). In Malaysia, similar constraints exist as public health spending remains below regional and global benchmarks. In 2023, public health expenditure was 2.83% of gross domestic product (GDP), and in 2022 it accounted for only 1.98% of GDP when adjusted for purchasing power parity, compared with the Organisation for Economic Co-operation and Development (OECD) average of 6.94%, highlighting substantially lower investment despite Malaysia’s upper-middle-income status (9). This financial burden makes managing myeloma a significant challenge, requiring a careful balance between treatment efficacy and affordability (8).

With the rising prevalence of MM in Malaysia and the limited access to newer therapies, reliance on international guidelines (1, 10) alone is insufficient for local needs. Therefore, these consensus guidelines were developed to provide a practical, resource-appropriate framework for care. Given the limited region-specific evidence, we adopted a consensus-based approach to ensure that the recommendations reflect current expert experience and real-world practice. By adapting international recommendations to Malaysia’s unique patient population, healthcare infrastructure, and available resources, these consensus guidelines aim to enhance clinical effectiveness and standardise care delivery across the country.

## Methods

### Consensus Development Process

An internal planning committee identified experts with extensive MM management experience and invited them via a formal email invite in January 2025. The invitation described the objectives, scope, and expected contribution to the consensus study. To ensure balanced representation, experts were selected from public, academic, and private institutions across Malaysia. The final panel comprised two steering committee members and a nine-member working group. All participants provided informed consent. This panel size falls within the optimal range for Delphi methodology (8 to 23 participants), ensuring sufficient expertise while enabling effective deliberation and consensus-building (11). The panel defined the study objectives and identified key issues and areas. Twelve areas were identified for inclusion in the Delphi consensus meeting, including diagnosis, treatment, and management of MM, namely: i) definitions of MM (symptomatic vs. asymptomatic); ii) diagnosis; iii) prognostic factors: disease staging and risk stratification model; iv) response criteria; v) transplant-eligible patients with newly diagnosed MM (NDMM); vi) transplant-ineligible (TIE)/deferred patients with NDMM; vii) treatment regimens for patients with renal dysfunction; viii) relapsed or refractory multiple myeloma (RRMM) early relapse (1 to 3 prior therapies); ix) RRMM late relapse (> 3 prior therapies); x) infection prevention in patients with MM; xi) treatment considerations for high-risk smouldering multiple myeloma (SMM); and xii) supportive care.

A targeted literature review was conducted to identify gaps in the management of MM globally and in Malaysia. PubMed and Google Scholar were searched to identify relevant articles published between January 2012 and June 2025, using predefined search terms provided in Supplementary Material 1. Studies were included if they were relevant to the diagnosis, risk stratification, treatment, and supportive care of MM; involved adult patients (≥ 18 years) with MM; reported clinical guidance, consensus recommendations, clinical trial data, real-world evidence, or expert review; and were published in English. Priority was given to recent publications, international guidelines, regional guidelines, landmark clinical trials or studies, and evidence considered clinically relevant to Malaysian or Asian practice. Preclinical or laboratory-based studies without direct clinical relevance, case reports, case series with sample size less than 20, conference abstracts, non-English publications, and opinion-based articles without any clinical recommendations were excluded. The evidence was then graded according to the Grading of Recommendations, Assessment, Development and Evaluations (GRADE) criteria (Supplementary Table 1) (12, 13).

Based on the findings of the literature review and the clinical experience of the core steering committee, 58 clinically relevant gap statements were developed and categorised into the domains described above. These statements, along with supporting evidence, were circulated to all panel members. Formal pilot testing was not conducted, as the voting statements were reviewed by the steering committee before circulation to the wider panel. The modified Delphi process allowed participants to provide feedback and seek clarification during the iterative rounds. In addition, we applied a two-tier resource classification, wherein “limited resource” refers to essential services that can substantially improve outcomes and are achievable with modest infrastructure and financial constraints. The “enhanced resource” refers to additional services that, while not mandatory in constrained settings, can further improve outcomes and expand available treatment options (14). This study was not registered on any public online platform.

### Consensus Voting and Data Collection

All 11 panel members participated in the modified Delphi process (15), involving anonymous electronic voting conducted via the SurveyMonkey platform. Each statement was assessed using a three-point Likert scale (“agree,” “neutral,” or “disagree”). Panellists selecting “neutral” or “disagree” were required to provide a written rationale for their response. This scale was selected to enhance the clarity of consensus, reduce participant fatigue, and simplify the interpretation of results. The use of this scale also minimises neutral or indecisive responses and facilitates a more efficient response process.

In alignment with the Conducting and REporting of DElphi Studies (CREDES) guidance, consensus was predefined as high consensus (≥ 80% agreement or disagreement), moderate consensus (55% to 79% agreement or disagreement), and low consensus (< 55% agreement or disagreement) (there is no universally accepted threshold for defining consensus) among panel members, and completion of all statement evaluations was mandatory. Following Round 1, a virtual meeting was conducted to discuss the voting results and revise statements that failed to reach ≥ 80% agreement. Statements that met the prespecified definition of consensus were also carried forward into Round 2 to confirm the stability of agreement and allow participants the opportunity to revise their ratings based on group feedback. Revised statements were circulated and revoted via a SurveyMonkey link during Round 2 (Figure 1).

## Results

The consensus exercise commenced on 29 October 2025 and progressed through all stages without delays. All 58 statements were included in Round 1 voting (29 October to 14 November 2025), followed by a three-day analysis period. In Round 1, 36 statements achieved high consensus, 18 achieved moderate consensus, and 4 achieved low consensus. A virtual meeting was held on 1 December 2025 to review and refine the Round 1 findings. Based on expert feedback, the 22 statements (Statements 2, 5, 7, 9, 13, 15, 17, 18, 22, 23, 24, 27, 28, 29, 30, 34, 36, 37, 42, 45, 47, and 49) with moderate or low consensus were modified and advanced to Round 2. In addition, two statements (Statements 1 and 55) that had already achieved high consensus in Round 1 were revisited in Round 2 following further deliberation and discussion among experts during the virtual meeting. Statement 1 originally listed bone lesions under both myeloma-defining events (MDEs) and additional MDEs, creating potential ambiguity and redundancy; experts therefore recommended that bone lesions be retained only under MDEs. Statement 55, as originally worded, implied that lenalidomide was the sole option for early intervention in selected patients with high-risk SMM. Based on evolving clinical evidence, experts recommended incorporating daratumumab monotherapy as an alternative in settings where it is clinically appropriate, feasible, and affordable. Both proposed revisions were discussed among the expert panel, and consensus was reached to submit the revised statements for another round of voting. Therefore, a total of 24 statements were circulated for Round 2 voting, which followed the same voting procedure and predefined consensus criteria as Round 1. Round 2 was conducted online and followed by a further three-day analysis period (4 to 8 December 2025). Following Round 2, all statements achieved a high level of consensus, defined as ≥ 80% agreement. The final list of consensus recommendations is presented in Supplementary Table 2.

### Definitions of MM (Symptomatic vs. Asymptomatic)

#### Symptomatic

In 2003, the International Myeloma Working Group (IMWG) introduced the acronym CRAB to summarise the cardinal clinical features of MM: hypercalcaemia, renal insufficiency, anaemia, and bone lesions (16). The CRAB criteria distinguish active, symptomatic MM from precursor disorders such as monoclonal gammopathy of undetermined significance (MGUS) and SMM. Based on the organs involved, patients with MGUS who develop monoclonal protein-related organ dysfunction are reclassified as having monoclonal gammopathy of clinical significance (MGCS) or monoclonal gammopathy of renal significance (MGRS) (1, 17).

A diagnosis of MM requires the presence of at least one MDE together with evidence of ≥ 10% clonal bone marrow plasma cells (BMPCs) or a biopsy-proven plasmacytoma. MDEs include traditional CRAB features as well as three biomarkers, collectively referred to as the SLiM criteria: clonal BMPCs ≥ 60%, serum free light chain ratio (sFLCr) ≥ 100 with involved free light chains (FLC) ≥ 100 mg/L, and > 1 focal lesion ≥ 5 mm on magnetic resonance imaging (MRI). Each SLiM biomarker carries approximately 80% risk of progression to symptomatic end-organ damage (1, 10). Table 1 summarises the IMWG diagnostic criteria used to distinguish symptomatic MM from other plasma cell disorders (18).

#### Asymptomatic

In most cases, MM originates from an asymptomatic premalignant stage known as MGUS. This phase is characterised by < 10% clonal BMPCs and an absence of MM-related symptoms. When the clonal plasma cell burden reaches 10% to 60% in the bone marrow, the condition is classified as SMM, which remains clinically asymptomatic and is typically recognised through standard clinical and laboratory assessment (1, 10). Although many patients with SMM remain stable for years and do not require treatment, all have a risk of progression to MM (1, 10, 17, 19). The progression rate varies widely—from months to years—according to individual risk factors, with high-risk patients requiring closer surveillance. Additionally, some individuals may transition from low- to high-risk SMM over time, necessitating adjustments in monitoring strategies (1). The risk of developing symptomatic MM (≥ 10% clonal BMPCs) is influenced by both tumour burden and the underlying cytogenetic abnormalities. Patients harbouring t(4;14), del(17p) or gain(1q) exhibit a higher risk of progression (10, 20–22). Importantly, asymptomatic patients who meet SLiM-CRAB criteria are considered to have active MM and should be managed accordingly (1, 10, 16, 18). Table 1 presents the recommendations for distinguishing between asymptomatic and symptomatic MM.

### Diagnosis

Monoclonal protein is detected in approximately 97% of cases, although it is not mandatory for MM diagnosis (18). The panel recommends serum protein electrophoresis (SPEP), serum immunofixation electrophoresis (SIFE), and serum free light chain (sFLC) testing as first-line investigations in patients with suspected MM. Diagnostic sensitivity is optimised when SPEP and SIFE are combined with sFLC testing (23–25). Routine urine screening for monoclonal protein is generally not recommended when serum-based tests are available, as it provides limited diagnostic or prognostic value (26, 27).

Conventional cytogenetic analysis has limited sensitivity due to low plasma cell proliferation and suboptimal metaphase yields. Fluorescence in situ hybridisation (FISH) overcomes this limitation and remains the standard method for detecting clinically relevant cytogenetic abnormalities (28). FISH sensitivity increases with plasma cell enrichment from bone marrow samples; however, the method can only identify chromosomal abnormalities that are predetermined, which represents a significant constraint. Advances in molecular techniques, including gene expression profiling (GEP) and next-generation sequencing (NGS), now allow broader characterisation, leading to the identification of driver mutations, oncogenic dependencies, structural variants, chromoplexy, and chromothripsis associated with clonal behaviour and disease progression (29). Although NGS enables more comprehensive detection of clinically relevant genetic abnormalities, many developing Asian countries, including Malaysia, rely on conventional cytogenetic techniques (G-banding and FISH) in accordance with current recommendations and available resources (29). However, in enhanced resource settings, the panel supports the use of bone marrow NGS to refine risk stratification and inform treatment decision-making, while acknowledging that it is not essential. Regarding imaging, low-dose whole-body computed tomography (CT) or positron emission tomography/CT (PET/CT) is preferred over conventional skeletal surveys (CSS) because of its superior sensitivity. MRI is particularly valuable in cases of suspected SMM, spinal involvement, and extramedullary disease. CSS should be reserved for settings in which advanced imaging modalities are unavailable (30, 31). Based on these considerations, recommendations for the diagnosis of MM are presented in Table 2.

### Prognostic Factors: Disease Staging and Risk Stratification Model

Accurate prognostication is essential due to marked heterogeneity in clinical outcomes (32). The International Staging System (ISS), based on serum β2-microglobulin level and albumin levels, remains widely used. The Revised International Staging System (R-ISS), incorporating serum lactate dehydrogenase and high-risk cytogenetic abnormalities detected by FISH, is currently the standard prognostic model for patients with NDMM (Table 3) (33, 34).

Despite its widespread use, the R-ISS has several limitations, including heterogeneity within stage II and inconsistent evaluation of chromosome 1 abnormalities (35). Several newer risk classification systems, including Second Revision of the International Staging System (R2-ISS) (35), Mayo Additive Staging System (MASS) (36), and Mayo Stratification of Myeloma and Risk-Adapted Therapy (mSMART) (37), have emerged to address some of the limitations of earlier models. Recent studies suggest that the MASS may offer complementary prognostic insights and improved practical applicability compared with mSMART and the R-ISS, with performance comparable to the R2-ISS (38, 39). However, available evidence is largely retrospective and population-specific, requiring further validation before routine clinical adoption. Consensus recommendations for staging and risk stratification of MM are presented in Table 3.

### Response Criteria

The IMWG response criteria provide a standardised framework for evaluating treatment efficacy (40). In parallel, an ongoing “cure vs. control” debate persists in MM management, questioning whether treatment should pursue an intensive multidrug strategy aimed at achieving a complete response (CR) or adopt a sequential disease-control approach that prioritises quality of life alongside overall survival (41, 42). Evidence suggests that when serum or urine immunofixation lags CR criteria despite BMPCs being < 5%, a repeat bone marrow biopsy assessment is not necessary and unlikely to alter management outside the context of clinical trials. Incorporation of this pragmatic approach into current IMWG CR criteria may help to reduce unnecessary invasive procedures for patients (43).

While achieving CR is an important therapeutic goal, it remains an imperfect surrogate for long-term outcomes (44). Minimal residual disease (MRD) assessment using next-generation flow or NGS offers greater sensitivity and has demonstrated strong prognostic value, detecting one malignant cell among 10^5^ normal cells (45–49). MRD testing is not yet widely accessible in Malaysia; however, in centres with advanced resources, it may be used for additional prognostic information. Treatment modification based solely on MRD status is not currently recommended outside clinical trials due to insufficient evidence (50, 51). The following recommendations outline the application of IMWG response criteria incorporating both standard response categories and the emerging role of MRD testing in enhanced resource settings (Table 4).

### Transplant-eligible Patients with NDMM

The management of transplant-eligible patients with NDMM has evolved substantially over the past decade, with significant advances in induction regimens, consolidation strategies, and post-transplant maintenance therapy (52). Across Asia, evidence demonstrates that triplet regimens are superior to doublet regimens, achieving higher remission rates and improved overall response rate (ORR), progression-free survival (PFS), and overall survival (OS) in both NDMM and RRMM patients (6, 53–56). Three drug regimens are recommended as the minimum induction therapy for transplant-eligible patients. These should include two different drug classes (PI and IMiD) and a corticosteroid. Bortezomib–lenalidomide–dexamethasone (VRd) (57), bortezomib–thalidomide–dexamethasone (VTd) (58), and carfilzomib–lenalidomide–dexamethasone (KRd) (59) are reasonable options. Although PI–IMiD triplet regimens are preferred, bortezomib–cyclophosphamide–dexamethasone (VCd) is also an acceptable induction option where IMiD-based therapy is not suitable or accessible (Table 5) (60). Also, concerns have been raised about the potential risk of cardiac toxicity associated with carfilzomib, and further data are required to better define its safety profile (10).

Quadruplet induction regimens incorporating anti-CD38 mAbs such as daratumumab plus VRd (DVRd), daratumumab plus VTd (DVTd), and isatuximab plus VRd (Isa-VRd) have demonstrated superior efficacy compared to triplet therapy, including higher ORRs, increased MRD negativity, and improved PFS and OS (10, 61, 62). However, clinical trials evaluating these regimens across Asia remain scarce due to regulatory constraints, operational challenges, and insufficient investment from the pharmaceutical industry, which restricts access to these novel agents and increases cost-related challenges. Hence, quadruplet regimens are recommended where regulatory approval, insurance coverage, and resources allow. The regimen selection should be individualised based on patient characteristics, disease attributes, and financial considerations (6).

For patients eligible for transplantation, induction therapy is typically followed by autologous stem cell transplantation (ASCT) consolidation and maintenance treatment with an IMiD such as lenalidomide (Table 5). Evidence from the Myeloma XI trial suggests that extending lenalidomide maintenance beyond three years leads to improved PFS. However, the magnitude of benefit appears to diminish over time, with minimal incremental advantage beyond four to five years following ASCT, particularly in patients who achieve MRD negativity (63). The financial burden, cumulative toxicity, and practicality of prolonged or indefinite lenalidomide therapy should therefore be carefully considered (10). Thalidomide may be considered as an alternative maintenance option in selected cases but is not recommended for patients with high-risk MM (64).

Despite the availability of novel agents, MM remains challenging to manage in the cases of aggressive relapse or multi-refractory disease, particularly when associated with high tumour burden and extramedullary involvement. Management in this clinical setting may require the use of conventional chemotherapy with novel agents in combination regimens. One such regimen is bortezomib, dexamethasone, thalidomide, cisplatin, doxorubicin, cyclophosphamide, and etoposide (VTD-PACE), a potent regimen that integrates novel agents with conventional chemotherapy and may be considered in this setting (1, 65, 66). However, it is often poorly tolerated and is associated with a high incidence of treatment-related adverse events (such as severe cytopenias, neuropathy, and thromboembolic complications) (67). Additionally, patients with ultra-high-risk (UHiR) MM also require early identification and intensified, individualised first-line treatment to achieve durable disease control. Established UHiR NDMM markers include ≥ 2 high-risk cytogenetic aberrations (double hit) and high-risk gene expression profiles, which are independent of a double hit and are associated with tumour proliferation (66, 68, 69). The OPTIMUM trial is the first study dedicated to UHiR NDMM and plasma cell leukaemia. The findings indicate that daratumumab, cyclophosphamide, bortezomib, lenalidomide, and dexamethasone (DCVRd) induction followed by ASCT and extended DVR(d) consolidation is highly effective in this setting (69). The recommendations for transplant-eligible patients with NDMM are presented in Table 5, and the approach to treating symptomatic NDMM is outlined in Figure 2. Treatment is determined by eligibility for ASCT and disease risk stratification.

### Transplant-ineligible/Deferred Patients with NDMM

In TIE NDMM, treatment selection is guided by patient-related factors, including age, overall fitness, frailty status, cytogenetic risk, comorbidities, quality of life, and cost or insurance coverage (Table 6). Frailty represents age-related decline in physiological reserve, compromising stress tolerance and associated with reduced chemotherapy tolerance, increased toxicity, treatment discontinuation, and mortality. The IMWG Frailty Score (IMWGFS), incorporating functional and clinical parameters, is the gold standard myeloma-specific geriatric assessment tool (70). Current international guidelines identified VRd and DRd (daratumumab, lenalidomide, and dexamethasone) as preferred frontline regimens for TIE patients, with regimen choice influenced by frailty status and treatment tolerability (1, 10, 71). Updated findings from the MAIA study, with a median follow-up exceeding five years, continue to demonstrate durable PFS and OS benefit with DRd compared with Rd, as frontline therapy for TIE patients (72). In settings where access to lenalidomide and anti-CD38 mAbs is limited, alternative bortezomibor alkylator-based regimens including VTd (bortezomib, thalidomide, and dexamethasone), VCd (bortezomib, cyclophosphamide, and dexamethasone), VMP (bortezomib, melphalan, and prednisone), and MPT (melphalan, prednisone, and thalidomide) remain reasonable options (10, 14, 73). For selected fit, non-frail TIE patients, quadruplet regimens such as DVRd and Isa-VRd can be considered as treatment options (10).

Following induction, ongoing maintenance therapy should be individualised with respect to agent, dose, and duration. Lenalidomide maintenance is recommended following induction with VRd, DRd, or DVRd. Thalidomide maintenance may be considered in patients treated with thalidomide-based induction therapy (74). Daratumumab and lenalidomide maintenance can be considered in selected standard-risk patients aiming to achieve deeper remission. For patients with high-risk myeloma, maintenance therapy with bortezomib plus lenalidomide or daratumumab plus lenalidomide may be considered where economically feasible (10). Consensus recommendations for transplant-ineligible patients with NDMM are presented in Table 6.

### Treatment Regimens for Patients with Renal Dysfunction

Renal impairment occurs in 20% to 40% of patients at diagnosis and significantly influences treatment selection (75, 76). Bortezomiband daratumumab-based regimens can be safely administered without dose adjustment, even in dialysis-dependent patients, whereas lenalidomide requires dose adjustment (1). Treatment strategies should aim to optimise disease control while minimising toxicity in this high-risk population. Evidence-based recommendations are provided in Table 7.

### Management of Relapsed and Refractory MM

Despite advances in frontline therapy, MM remains largely incurable, with the majority of patients experiencing disease relapse requiring subsequent lines of treatment (77, 78). Treatment selection at relapse must consider multiple factors, including the nature and timing of prior therapies, depth and duration of previous responses, fitness status, disease biology, and available resources (79). With respect to pharmacologic treatment, a triplet regimen incorporating at least two novel agents to which the patient is not refractory should be considered. In eligible patients, ASCT should be considered for those who are transplant-naïve or who achieved a sustained remission of at least 36 months or longer following their first ASCT with maintenance therapy (10).

Cellular immunotherapy has also advanced rapidly, with approved B-cell maturation antigen (BCMA)-targeted chimeric antigen receptor (CAR) T-cell therapies and bispecific T-cell engagers (BiTEs) demonstrating substantial clinical efficacy. However, achieving durable remission across a broader patient population remains challenging (80). The Phase 3 CARTITUDE-4 trial demonstrated that cilta-cel, a BCMA-directed CAR-T therapy, reduced the risk of disease progression or death compared with standard care in lenalidomide-refractory patients who had received one to three prior lines of therapy (81). Similarly, the MajesTEC-3 trial showed that teclistamab (a bispecific antibody engaging CD3 on T cells and BCMA on myeloma cells) combined with daratumumab resulted in significantly longer PFS than daratumumab, pomalidomide, and dexamethasone (DPd) or daratumumab, bortezomib, and dexamethasone (DVd) groups (82). In addition, the combination of belantamab mafodotin, bortezomib, and dexamethasone (BVd) is also emerging as a potential treatment option for both lenalidomide-exposed and lenalidomide-refractory MM (83). However, the optimal sequencing of these agents remains an evolving area, particularly in resource-variable settings such as Malaysia, where access to newer therapies is limited (84). The following statements provide evidence-based recommendations in this setting across different lines of therapy, considering both resource-enhanced and resource-constrained settings (Table 8). The overall treatment approach to RRMM is outlined in Figure 3.

### Management of Late Relapse in RRMM (≥ 3 Prior Therapies)

Patients with MM who have received three or more prior lines of therapy represent a particularly challenging population, often characterised by triple-class refractory disease (refractory to PIs, IMiDs, and anti-CD38 mAbs) and limited treatment options (85, 86). Treatment outcomes in heavily pretreated patients have been suboptimal, with a median PFS of three to five months and OS < 13 months (87–89). The therapeutic landscape has been transformed by the emergence of novel immunotherapeutic approaches, including CAR-T cell therapies and bispecific T-cell engager antibodies targeting BCMA as well as non-BCMA antigens such as GPRC5D, which have shown remarkable efficacy in this heavily pretreated population (1, 10, 90). Nevertheless, access to these innovative therapies remains limited in many regions due to infrastructure requirements, regulatory approvals, and cost considerations. As a result, alternative treatment strategies are required in resource-constrained environments (14). The following statements provide consensus recommendations for the management of late relapse, addressing both cutting-edge immunotherapies available in enhanced resource settings and practical alternatives in limited-resource contexts, while emphasising the importance of clinical trial participation whenever feasible (Table 9).

### Infection Prevention in Patients with MM

Infectious complications remain a leading cause of morbidity and mortality in patients with MM, accounting for approximately 10% of premature deaths (91). Prolonged exposure to novel immunotherapies, including anti-CD38 mAbs and, more recently, bispecific antibodies targeting CD3 and BCMA, GPRC5D, or FCRH5, is associated with an increased risk of infection, partly attributable to hypogammaglobulinaemia (92). Recent evidence demonstrates that proactive infection prevention strategies, including antimicrobial prophylaxis, immunoglobulin replacement therapy, and vaccination, can significantly reduce infectious complications and improve outcomes (91, 93). High rates of preexisting hypogammaglobulinaemia have been observed even in early lines of therapy. The use of intravenous immunoglobulin (IVIG) during immune-based treatments has been shown to reduce the annual rate of all-grade infections by 40% (93). The panel recommends that patients with recurrent infections undergo an assessment of immune status, including a detailed infection history and laboratory evaluation of immune parameters to identify candidates for early IVIG therapy. Although the optimal IVIG dosing schedule remains undefined, the primary goal is prevention of further infections. The following statements provide evidence-based recommendations for implementing comprehensive infection prevention strategies throughout the treatment continuum, from initial diagnosis to relapsed and refractory disease (Table 10).

### Treatment Considerations for High-risk SMM

Traditionally, SMM has been managed with active surveillance. However, evidence suggests that selected high-risk patients may benefit from early therapeutic interventions (1, 94, 95). Contemporary risk stratification models, including the updated Mayo Clinic criteria (20/20/20) and the Spanish PETHEMA model, enable identification of patients at the highest risk of progression, with selected cohorts demonstrating progression rates exceeding 50% at two years (96, 97). Early treatment with single-agent lenalidomide or daratumumab monotherapy has been shown to significantly delay progression to active myeloma in carefully selected patients with high-risk SMM (1). In the recently published AQUILA trial, subcutaneous daratumumab monotherapy was associated with a significantly reduced risk of progression to active MM or death, compared with active monitoring, and demonstrated a favourable OS trend, although longer follow-up is required to confirm a definitive OS benefit (98). The evidence is still evolving, and treatment of SMM remains an area of debate. Most clinicians prefer active surveillance due to concerns about overtreatment and the costs associated with long-term therapy. Regardless of the approach, patients should be monitored closely to detect disease progression and manage potential treatment-related toxicities (95, 99). Additional randomised trials are required to determine whether early treatment of SMM in the modern era provides an overall clinical benefit (100). Table 11 outlines the consensus-based treatment recommendations for patients with high-risk SMM.

### Supportive Care

Bone disease is one of the most common and debilitating complications of MM, occurring in approximately 80% of patients (101). Skeletal-related events (SREs), including pathological fractures, radiation to bone, surgery to bone or spinal cord compression, occur in up to 50% of patients within the first two years of diagnosis without preventive intervention (102). Bone-targeting agents, including bisphosphonates and the receptor activator of nuclear factor kappa-B ligand (RANKL) inhibitor such as denosumab, have demonstrated significant efficacy in reducing SREs, improving bone pain, and potentially exerting anti-myeloma effects (1, 103). For patients receiving bisphosphonates, regular renal function monitoring is recommended. Additionally, a dental examination should be performed before initiating therapy, and patients should be monitored for signs of osteonecrosis of the jaw throughout treatment (1). A subgroup analysis suggests that denosumab is a viable alternative for Asian patients with NDMM and lytic bone disease (104). However, it should also be noted that severe hypocalcaemia is an uncommon but potentially life-threatening adverse effect of denosumab. Early recognition, careful monitoring of calcium levels, and timely calcium replacement are essential for effective management (105). Beyond pharmacological interventions, comprehensive management of myeloma bone disease requires a multidisciplinary approach that incorporates radiation therapy, orthopaedic surgical intervention, and rehabilitation services (58). Supportive care should be individualised based on patient characteristics, treatment response, and the agent used (Table 12).

## Discussion

This expert consensus provides a comprehensive, evidence-based and evolving framework for the diagnosis and management of MM tailored to the Malaysian healthcare context.

The IMWG SLiM-CRAB criteria remain central to distinguishing symptomatic from asymptomatic disease and guiding treatment initiation (1, 10). However, in resource-constrained settings such as Malaysia, access to specialised tests and advanced imaging modalities may be limited by cost and logistical factors. Superior resolution of cytogenetic abnormalities can be achieved with NGS; however, conventional cytogenetics and FISH remain the mainstay of diagnostic evaluation in many centres (29, 30). Treatment strategies for NDMM reflect substantial therapeutic advances over the past decade. In transplant-eligible patients, triplet induction regimens remain the minimum accepted standard, supported by consistent improvements in response rates, PFS, and OS (6, 53–56). Quadruplet regimens incorporating anti-CD38 mAbs have demonstrated superior depth of response, higher rates of MRD negativity, and improved survival outcomes (10, 61, 62). However, practical barriers such as regulatory approval, treatment cost, and unequal access to novel agents limit universal implementation across Asian healthcare systems (6). In TIE patients, treatment decisions are guided by age, overall fitness and degree of frailty, cytogenetic risk profile, coexisting illnesses, quality of life, and financial considerations (10). Renal impairment is also a frequent and clinically significant complication of MM that influences treatment selection and prognosis (75, 76). Evidence supports the use of bortezomib- and daratumumab-based regimens without dose adjustment, even in patients with severe renal dysfunction or dialysis dependence (1).

The emergence of cellular and immune-based therapies, including CAR-T cell therapy and bispecific antibodies targeting BCMA, has significantly improved outcomes in heavily pretreated populations (14). Nonetheless, access to these therapies remains limited in many regions; therefore, exploring alternative strategies and promoting participation in clinical trials in resource-constrained settings is essential (91, 92). Disease-related immune dysfunction and treatment-related immunosuppression, particularly with anti-CD38 mAbs and bispecific therapies, increase susceptibility to infection through mechanisms including hypogammaglobulinaemia (93). Evidence supports the use of antimicrobial prophylaxis, IVIG therapy in patients with recurrent infections, and vaccination as integral components of comprehensive MM care (91, 92). Myeloma-related bone disease affects most patients and significantly contributes to morbidity. Early use of bone-targeting agents (bisphosphonates or denosumab) reduces SREs and improves quality of life. Optimal outcomes require a multidisciplinary approach integrating systemic therapy, radiation, orthopaedic intervention, and rehabilitation (1, 104, 106).

### Strengths and Limitations

A key strength of this consensus lies in its explicit recognition of resource variability within the Malaysian healthcare system. Unlike international guidelines that often presume universal access to cutting-edge therapies, these recommendations stratify treatment options according to resource availability. The rigorous methodology and evidence-based implementable recommendations ensure that clinicians can provide optimal care within their institutional capabilities while maintaining awareness of emerging therapeutic options that may become available in enhanced resource settings. However, several key limitations should be noted, including potential selection bias in both the recruitment of the expert panel and the literature reviewed. In addition, clinical data on emerging novel agents within the Asian population remain limited. As frontline myeloma treatment continues to advance rapidly, this guidance must be viewed alongside newer clinical and real-world evidence published after the panel’s discussions.

## Conclusion

These recommendations provide an adaptable, evidence-based framework for MM management in Malaysia, aligning international standards with real-world practice constraints. Continued efforts to improve access to advanced diagnostic modalities, novel therapies, and clinical trial participation will be essential to further enhance patient outcomes.

## Supplementary Information



## Figures and Tables

**Figure 1:**
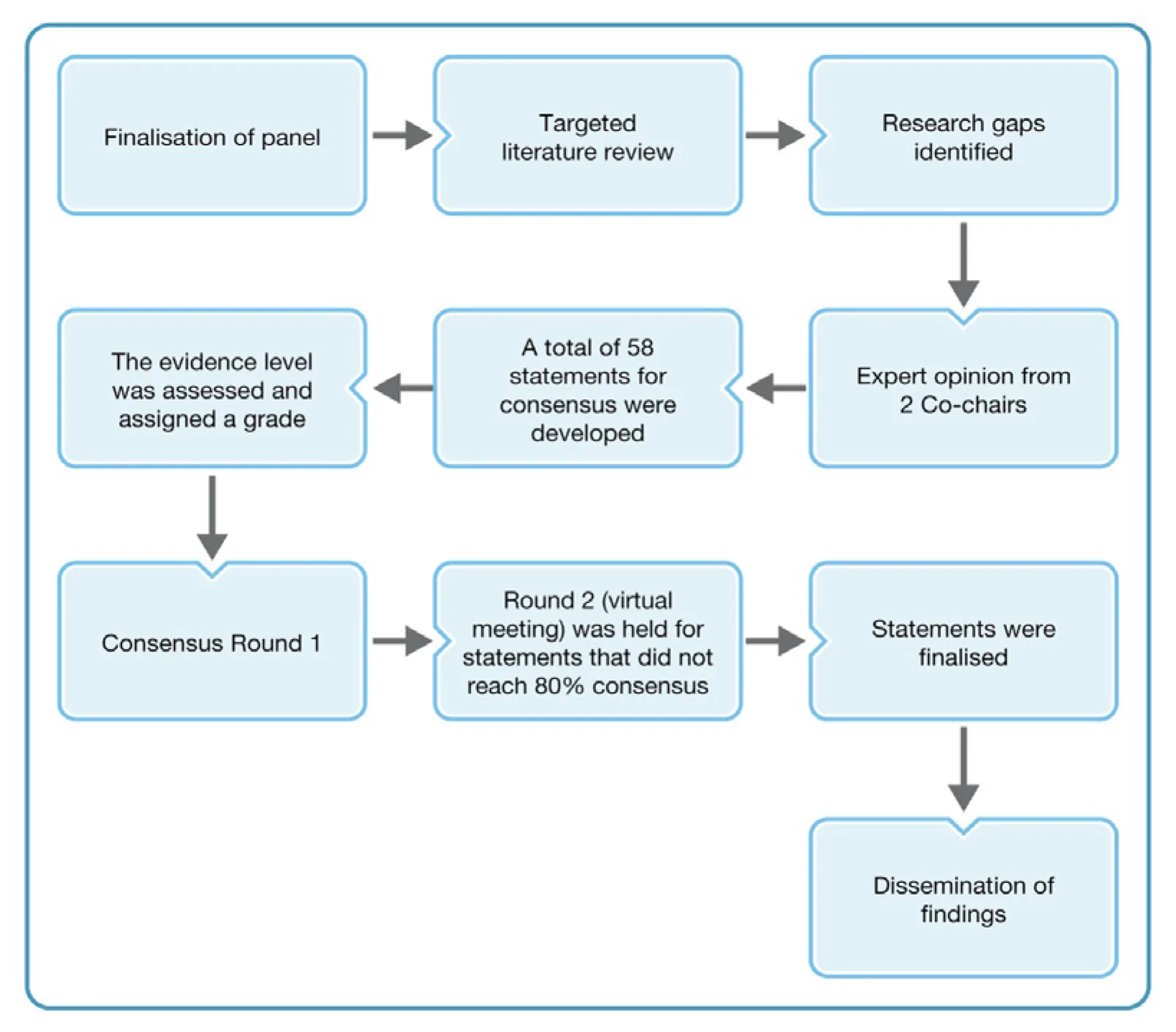
Overview of consensus process. *The only exceptions were Statements 1 and 55, which, despite reaching the predefined consensus threshold in Round 1, were revised based on expert recommendations and submitted for Round 2 voting.

**Figure 2:**
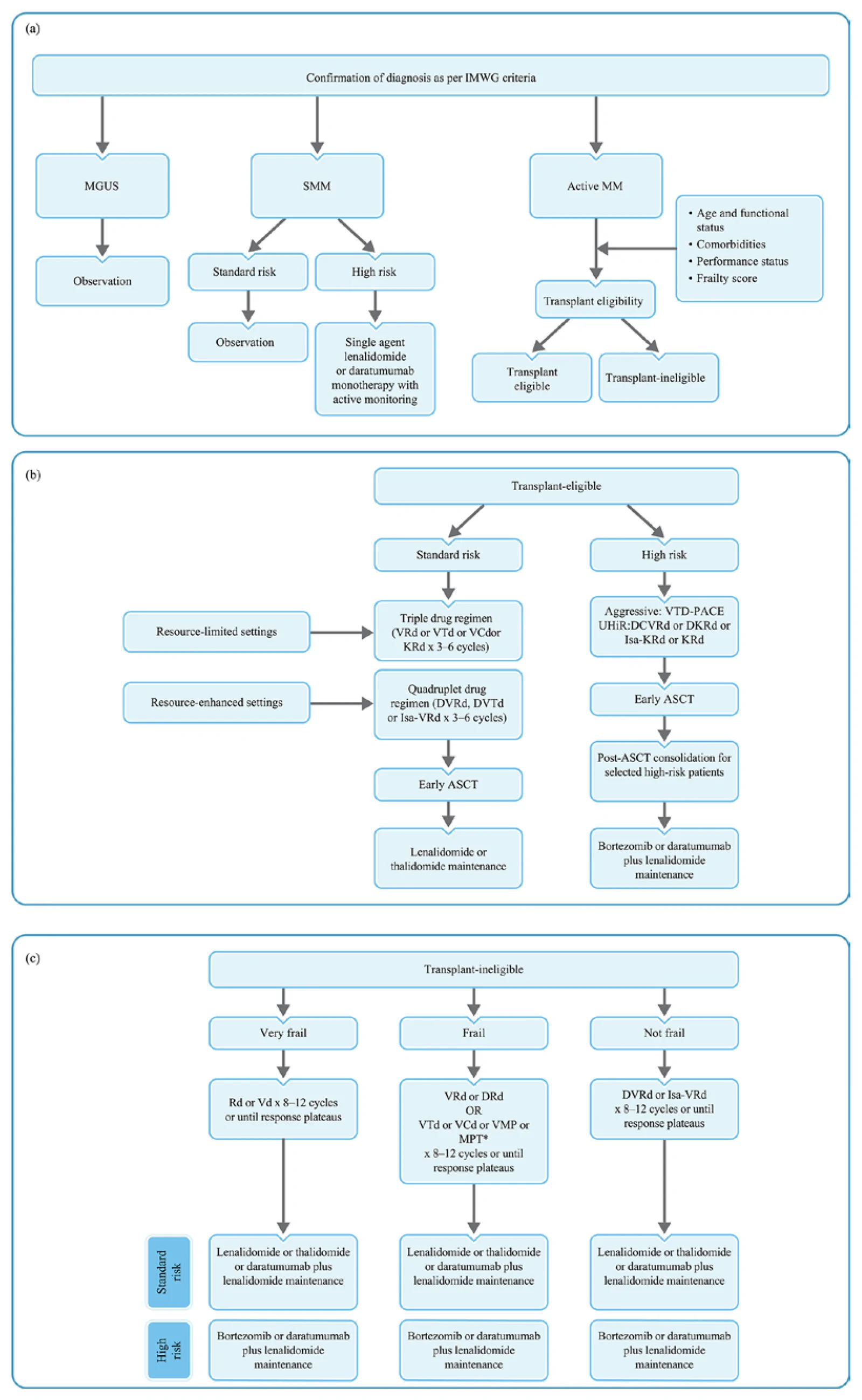
Treatment algorithm for NDMM management. ASCT = autologous stem cell transplantation; DCVRd = daratumumab, cyclophosphamide, bortezomib, lenalidomide, dexamethasone; DKRd = daratumumab, carfilzomib, lenalidomide, dexamethasone; DRd = daratumumab, lenalidomide, dexamethasone; DVRd = daratumumab, bortezomib, lenalidomide, dexamethasone; DVTd = daratumumab, bortezomib, thalidomide, dexamethasone; IMWG = International Myeloma Working Group; Isa-KRd = isatuximab, carfilzomib, lenalidomide, dexamethasone; Isa-VRd = isatuximab, bortezomib, lenalidomide, dexamethasone; KRd = carfilzomib, lenalidomide, dexamethasone; MGUS = monoclonal gammopathy of undetermined significance; MM = multiple myeloma; MPT = melphalan, prednisone, thalidomide; Rd = lenalidomide, dexamethasone; SMM = smouldering multiple myeloma; UHiR = ultra-high-risk; VCd = bortezomib, cyclophosphamide, dexamethasone; Vd = bortezomib, dexamethasone; VMP = bortezomib, melphalan, prednisone; VRd = bortezomib, lenalidomide, dexamethasone; VTd = bortezomib, thalidomide, dexamethasone; VTD-PACE = bortezomib, thalidomide, dexamethasone, cisplatin, doxorubicin, cyclophosphamide, etoposide. *Regimens recommended for resource-limited settings.

**Figure 3:**
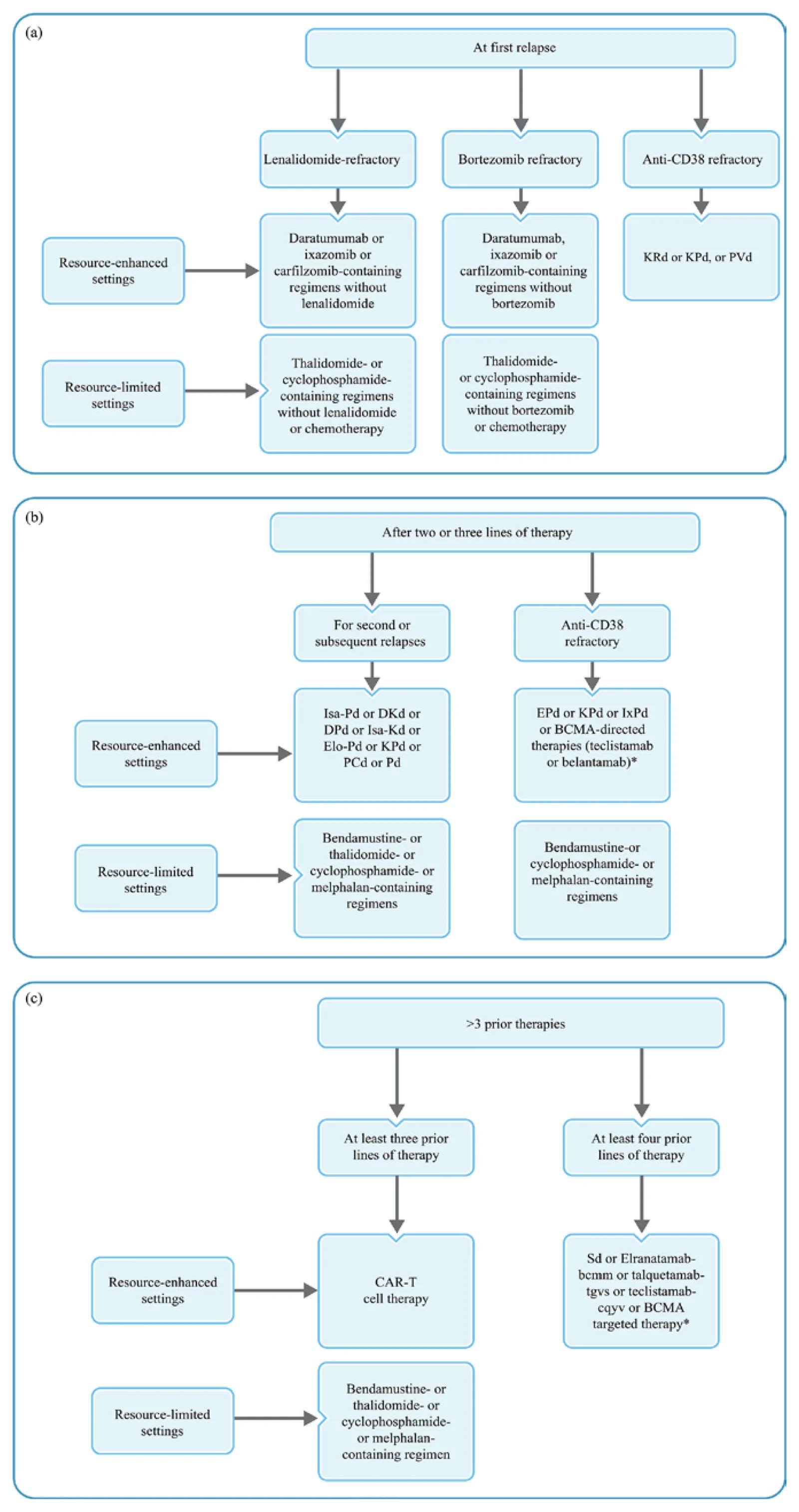
Treatment algorithm for RRMM management. BCMA = B-cell maturation antigen; CAR-T = chimeric antigen receptor T-cell; CD38 = cluster of differentiation 38; DKd = daratumumab, carfilzomib, dexamethasone; DPd = daratumumab, pomalidomide, dexamethasone; EPd = elotuzumab, pomalidomide, dexamethasone; Elo-Pd = elotuzumab, pomalidomide, dexamethasone; Isa-Kd = isatuximab, carfilzomib, dexamethasone; Isa-Pd = isatuximab, pomalidomide, dexamethasone; IxPd = ixazomib, pomalidomide, dexamethasone; KPd = carfilzomib, pomalidomide, dexamethasone; KRd = carfilzomib, lenalidomide, dexamethasone; PCd = pomalidomide, cyclophosphamide, and dexamethasone; Pd = pomalidomide, dexamethasone; PVd = pomalidomide, bortezomib, dexamethasone; Sd = selinexor, dexamethasone *Inclusion of agents such as teclistamab, talquetamab, and belantamab reflects evidence-based recommendations; however, availability, regulatory approval, and reimbursement may differ across regions. Implementation should be adapted to local access and clinical feasibility

**Table 1:** Consensus recommendations for symptomatic or asymptomatic MM.

Statement	Recommendation	Grade of evidence	Consensus
**Symptomatic**			
1	The diagnosis of symptomatic MM requires: Clonal BMPCs^a^ ≥ 10% or a biopsy-proven plasmacytoma, and evidence of any one or more of the following MDEs: Increased calcium levels (> 2.75 mmol/L) Renal insufficiency (creatinine > 177 μmol/L or creatinine clearance < 40 mL/min) Anaemia (haemoglobin < 10 g/dL or ≥ 2 g/dL below the lower limit of normal) ≥ 1 osteolytic bone lesions identified through skeletal radiography, CT, or FDG-PET/CT Additional MDEs include (SLiM features): Clonal BMPCs ≥ 60% sFLCr of ≥ 100 with an involved FLC concentration of at least 100 mg/L More than one focal lesion ≥ 5 mm detected on MRI	High	Round 1: 82% Round 2: 100%
**Asymptomatic**			
2	It is characterised by the presence of serum monoclonal protein levels of ≥ 30 g/L or Bence Jones protein levels of ≥ 500 mg/24 h, and/or clonal BMPCs comprising 10% to 60% of the marrow. SMM is distinguished from symptomatic MM by the absence of MDEs or amyloidosis.	High	Round 1: 64% Round 2: 100%
3	At diagnosis, BMPCs > 20%, M protein > 20 g/L, and sFLCr > 20 are key variables for risk stratification. Patients presenting with ≥ 2 of these risk factors are considered to have a high risk of 50% to 80% progression to symptomatic MM within two years from diagnosis.	High	Round 1: 82%

BMPCs = bone marrow plasma cells; CT = computed tomography; FDG-PET/CT = fluorodeoxyglucose positron emission tomography/computed tomography; FLC = free light chains; M = monoclonal; MDEs = myeloma-defining events; MM = multiple myeloma; MRI = magnetic resonance imaging; PET = positron emission tomography; SMM = smouldering multiple myeloma; sFLCr = serum free light chain ratio

a If bone marrow has less than 10% clonal plasma cells, then more than one bone lesion is required to make the diagnosis of MM

**Table 2:** Consensus recommendations for diagnosis of MM.

Statement	Recommendation	Grade of evidence	Consensus
4	Diagnosis of MM should include testing for M proteins using a combination of SPEP, SIFE, and the sFLC assay when the disease is clinically suspected. At the time of initial diagnosis, bone marrow studies should include chromosome analysis using FISH performed on plasma cells obtained from bone marrow aspiration. The FISH panel, used for prognostic assessment, should evaluate for abnormalities such as del(13), del(17p13), t(4;14), t(11;14), t(14;16), t(14;20), 1q21 gain/amplification, and 1p deletion. This information helps define the biological subtype of the disease, guides prognostic evaluation, and supports the determination of eligibility for clinical trials.	High	Round 1: 100%
5	To evaluate treatment response, M protein levels^a^ should be monitored 1–3 monthly during therapy using SPEP and sFLC assays, and every three to four months once therapy has ended. The sFLC assay is valuable, especially for patients without a measurable M protein when the FLCr is abnormal, or the involved FLC level is ≥ 100 mg/L. Additionally, urine protein electrophoresis may be performed every three to six months to monitor urinary M protein levels.	High	Round 1: 45% Round 2: 91%
6	Diagnosis of bone involvement in MM is best achieved using low-dose WB-CT or PET/CT, which offers superior sensitivity compared to CSS. MRI is particularly valuable for evaluating patients with suspected SMM as it identifies focal marrow lesions before osteolytic alterations, evaluates extramedullary disease, assesses spinal cord compression, and provides detailed imaging of symptomatic regions. CSS should be reserved for scenarios where advanced imaging options are not accessible.	High	Round 1: 82%
7	In enhanced resource settings, NGS of bone marrow can be performed to identify significant genetic abnormalities, providing crucial insights for improved risk stratification. While NGS is not mandatory, it is beneficial when available and can enhance treatment decision-making.	Moderate	Round 1: 36% Round 2: 100%

CSS = conventional skeletal surveys; FISH = fluorescence in situ hybridisation; FLC = free light chain; FLCr = free light chain ratio; MM = multiple myeloma; MRI = magnetic resonance imaging; NGS = next-generation sequencing; PET/CT = positron emission tomography/computed tomography; sFLC = serum free light chain assay; SIFE = serum immunofixation electrophoresis; SMM = smouldering multiple myeloma; SPEP = serum protein electrophoresis; WB-CT = whole-body computed tomography.

a Considered to be measurable if it is ≥ 10 g/L in the serum and or ≥ 200 mg/day in the urine.

**Table 3:** Consensus recommendations for staging and risk stratification.

Statement	Recommendation	Grade of evidence	Consensus
8	Those with active MM can be staged using ISS. The ISS identifies three stages based on serum β-2 microglobulin and serum albumin levels. The R-ISS further incorporates prognostic information from LDH levels and high-risk chromosomal abnormalities, including t(4;14), t(14;16), and 17p13 deletion, as detected by FISH. Other staging systems, such as MASS or mSmart, can also be considered (Grade of evidence: moderate)	High	Round 1: 100%
9	High-risk NDMM is defined by the presence of at least one of the following abnormalities: del(17p), with a cutoff of > 20% clonal fraction, and/or TP53 mutation An IgH translocation including t(4;14), t(14;16), or t(14;20) along with 1q+ and/or del(1p32) Monoallelic del(1p32) along with 1q+ or biallelic del(1p32) β2 microglobulin ≥ 5.5 mg/L with normal creatinine (< 106 μmol/L) Patients presenting with ≥ 2 high-risk cytogenetic abnormalities are considered to have a very high risk of disease progression or relapse	High	Round 1: 73% Round 2: 100%
10	Patients with relapsed MM are considered at high risk of further progression or relapse if they meet any of the following criteria: Disease relapse within 2 years of initial therapy when transplant and maintenance were used Disease relapse within 18 months when treated with non–transplant-based therapy Acquisition of high-risk cytogenetic abnormalities, such as 1q gain/1q21 amplification and/or del(17p)/TP53 mutation Presence of extramedullary disease at relapse and/or circulating plasma cells^a^	High	Round 1: 100%

FISH = fluorescence in situ hybridisation; IgH = immunoglobulin heavy chain; ISS = International Staging System; LDH = lactate dehydrogenase; MASS = Mayo Additive Staging System; MM = multiple myeloma; mSMART = Mayo Stratification of Myeloma and Risk-Adapted Therapy; NDMM = newly diagnosed multiple myeloma; R-ISS = Revised International Staging System; TP53 = tumour protein p53.

a Presence of ≥ 5% of plasma cells in circulation is defined as plasma cell leukaemia.

**Table 4:** Consensus recommendations for response criteria.

Statement	Recommendation	Grade of evidence	Consensus
11	As per IMWG response criteria (2016), MM is categorised into four levels: sCR^a^, CR^b^, VGPR^c^, and PR^d^; which helps assess and classify responses to treatment in clinical trials and routine practice, helping to ensure consistent reporting and comparison of treatment efficacy. Additional categories, such as MR^e^, PD^f^, and SD^g^, are also crucial for accurate assessment of treatment outcomes.	High	Round 1: 91%
12	The MM response criteria are recommended for assessing disease status in patients with MM. If some, but not all, criteria are met at any response level, the disease status should be downgraded to the next lower level of response category.	High	Round 1: 100%
13	In enhanced resource settings, when MRD assessment is available, sustained MRD-negative status may be verified by confirming bone marrow negativity using NGF or NGS techniques with a minimum sensitivity of 1 in 10^5^ nucleated cells or higher, with repeat evaluations performed at least one year apart.	High	Round 1: 45% Round 2: 91%

CR = complete response; IMWG = International Myeloma Working Group; MM = multiple myeloma; MR = minimal response; MRD = minimal residual disease; NGS = next-generation sequencing; NGF = next-generation flow; PD = progressive disease; PR = partial response; sCR = stringent complete response; SD = stable disease; VGPR = very good partial response.

a Requires normal FLCr, no clonal cells in bone marrow biopsy by immunohistochemistry, specific kappa/lambda ratio criteria apply.

b Negative M protein in serum and urine; disappearance of soft tissue plasmacytomas; less than 5% plasma cells in bone marrow aspirate; if the bone marrow is 5% plasma cells but positive serum/urine immunofixation, then a repeat test is needed.

c Detectable M protein in serum and urine, at least 90% reduction in serum M protein plus urine M protein level.

d At least 50% reduction in serum M protein, at least 90% reduction in 24-hour urinary M protein, and at least 50% size reduction of soft tissue plasmacytomas.

e At least 25% but less than 49% reduction in serum M protein, 50% to 89% reduction in 24-h urine M protein, or at least 50% increase in the size of soft tissue plasmacytomas (if present at baseline).

f Any one of the following four criteria applies: 25% increase from the lowest confirmed response value with (a) serum M protein absolute increase of ≥ 5 g/L; (b) urine M protein absolute increase of ≥ 200 mg/24 hours; (c) in patients without measurable serum and urine M protein: difference between involved and uninvolved FLC levels must increase by >10 mg/dL, BMPC percentage increase must be ≥ 10% of baseline status, appearance of new lesion(s), or ≥ 50% increase of > 1 lesion, or ≥ 50% increase in longest diameter of previous lesion > 1 cm in short axis; (d) at least 50% increase in circulating plasma cells (minimum 200 cells/μL).

g Not meeting criteria for sCR, CR, VGPR, PR, MR, or PD.

**Table 5:** Consensus recommendations for transplant-eligible patients with NDMM.

Statement	Recommendation	Grade of evidence	Consensus
**Transplant considerations**			
14	The first step in treating patients with NDMM is to assess their eligibility for high-dose therapy and ASCT, taking into account factors such as age and comorbidities. Advanced age and renal dysfunction, however, are not absolute contraindications to transplant. Therefore, early referral to an HCT centre for evaluation of transplant eligibility is recommended if the patient is willing to undergo ASCT.	High	Round 1: 82%
**Induction therapy**			
15	Three drug regimens should be the preferred minimum induction option for transplant-eligible patients. These triplet regimens should include two different drug classes (PI and IMiD) and a corticosteroid. In enhanced resource settings, where available, a quadruplet regimen can be considered as initial therapy based on improved RR, DpR, and PFS. Quadruplet regimens should include an anti-CD38 mAb, two different drug classes (PI and IMiD), and a corticosteroid. Frailty assessment should be performed in older adults, with consideration of dose modifications, particularly steroids, based on functional status and age.	High	Round 1: 73% Round 2: 91%
16	Doublet regimens are not recommended for transplant-eligible candidates; they are reserved for patients who are TIE with poor performance or frail status. A third drug may be added once the patient’s performance status improves.	High	Round 1: 100%
17	First-line three drug regimens options for transplant-eligible patients are VRd, VTd, VCd, and KRd.	High	Round 1: 73% Round 2: 100%
18	In enhanced resource settings, first-line anti-CD38-containing regimens for transplant-eligible patients are DVRd, DVTd or Isa-VRD. Induction with three to four cycles of the regimen is recommended.	High	Round 1: 64% Round 2: 100%
19	VTD-PACE may be considered for patients who are transplant-eligible with aggressive MM, such as those with extramedullary disease or plasma cell leukaemia. Other treatment options for high-risk MM in transplant-eligible patients include DCVRd for ultra-high-risk MM (i.e. ≥ 2 high-risk cytogenetic aberrations or plasma cell leukaemia). Additional options include DKRd, Isa-KRd, and KRd with or without tandem autologous or allogeneic HSCT, particularly for plasma cell leukaemia.	High	Round 1: 82%
**Consolidation and maintenance therapy**			
20	Early ASCT is recommended for eligible NDMM patients following three to six cycles of induction therapy; selected high-risk patients, especially those not achieving CR after their initial transplant, may be considered for additional consolidation cycles, especially in patients with high-risk cytogenetic abnormalities and ultra-high-risk MM.	High	Round 1: 91%
21	Consolidation with CAR T-cell therapy is not widely recommended following first-line therapy outside of clinical trial settings.	High	Round 1: 100%
22	Maintenance therapy with lenalidomide is recommended for a duration of two to four years as the standard approach, with consideration of longer treatment on a case-by-case basis depending on tolerability and toxicities. Thalidomide may be considered as an alternative depending on tolerability, access, and concerns regarding second malignancies associated with lenalidomide.	High	Round 1: 73% Round 2: 100%
23	Two-drug maintenance therapy can be considered for high-risk MM, and the options can be bortezomib plus lenalidomide or daratumumab plus lenalidomide.	High	Round 1: 64% Round 2: 91%
24	Thalidomide maintenance therapy may be a possible approach for standard-risk patients, showing significantly prolonged median PFS compared to no maintenance therapy. Its primary adverse effect is peripheral neuropathy, but advantages include the low risk of neutropenia, minimal adverse impact on stem cell harvest, and the fact that dose adjustments are not necessary in patients with renal impairment. However, thalidomide maintenance is associated with inferior PFS and OS in patients with unfavourable cytogenetics or high-risk MM.	High	Round 1: 73% Round 2: 91%
**Stem cell harvest and stem cell mobilisation**			
25	Stem cell toxins, such as nitrosoureas or alkylating agents, can reduce stem cell reserves. Regimens containing these agents, particularly melphalan, should be avoided in patients who may be candidates for HSCT until stem cells are harvested. If HSCT is delayed, stem cells should be collected and stored. It is recommended to harvest peripheral blood stem cells after four to six cycles of therapy, before prolonged exposure to lenalidomide and/or daratumumab, if HSCT is being considered.	Moderate	Round 1: 100%
26	Stem cell mobilisation should be performed with G-CSF, with or without prior chemotherapy using cyclophosphamide at a dose range of 1.5 to 4 g/m^2^. Additionally, plerixafor, a selective and reversible CXCR4 antagonist, should be considered to decrease mobilisation failure by inhibiting HSC adhesion to the marrow and promoting their circulation.	Moderate	Round 1: 82%
27	Stem cell mobilisation may be reduced after induction with lenalidomide- or daratumumab-containing regimens. Early stem cell mobilisation after 2 to 4 cycles of induction therapy is encouraged. An upfront plerixafor strategy may be considered in patients at high risk of poor mobilisation, particularly in enhanced resource centres.	High	Round 1: 64% Round 2: 100%

ASCT = autologous stem cell transplant; CAR T-cell = chimeric antigen receptor T-cell; CD38: Cluster of differentiation 38; CR = complete response; CXCR4 = C-X-C chemokine receptor type 4; DCVRd = daratumumab, cyclophosphamide, bortezomib, lenalidomide, and dexamethasone; DKRd = daratumumab, carfilzomib, lenalidomide, and dexamethasone; DpR = depth of response; DVRd = daratumumab, bortezomib, lenalidomide, and dexamethasone; DVTd = daratumumab, bortezomib, thalidomide, and dexamethasone; G-CSF = granulocyte colony-stimulating factor; HCT = haematopoietic cell transplantation; HSC = haematopoietic stem cell; HSCT = haematopoietic stem cell transplantation; IMiD = immunomodulatory drug; Isa-KRd = isatuximab, carfilzomib, lenalidomide, and dexamethasone; Isa-VRD = isatuximab, bortezomib, lenalidomide, and dexamethasone; KRd = carfilzomib, lenalidomide, and dexamethasone; MM = multiple myeloma; NDMM = newly diagnosed multiple myeloma; OS = overall survival; PFS = progression-free survival; PI = proteasome inhibitor; RR = response rate; TIE = transplant-ineligible; VCd = bortezomib, cyclophosphamide, and dexamethasone; VRd = bortezomib, lenalidomide, and dexamethasone; VTd = bortezomib, thalidomide, and dexamethasone; VTD-PACE = bortezomib, thalidomide, dexamethasone, cisplatin, doxorubicin, cyclophosphamide, and etoposide.

**Table 6:** Consensus recommendations for transplant-ineligible patients with NDMM.

Statement	Recommendation	Grade of evidence	Consensus
**Initial treatment**			
28	For TIE patients, first-line therapy should be individualised based on frailty, myeloma risk profile, and financial affordability. Based on published evidence, VRd or DRd is recommended as first-line therapy, while DVRd or Isa-VRd may offer an advantage in fitter patients. Rd or Vd can be considered for selected very frail patients. Induction therapy should be continued for 8 to 12 cycles or until response plateaus.	High	Round 1: 73% Round 2: 91%
29	For TIE patients with limited resources, the combinations of VTd, VCd, VMP, and MPT can be used as an alternative induction therapy. The respective regimen can be dose-adjusted for frail patients.	High	Round 1: 64% Round 2: 100%
**Maintenance therapy**			
30	In TIE patients, those who have received VRd, DRd, or DVRd as induction therapy should be followed by lenalidomide maintenance. Daratumumab and lenalidomide maintenance can be considered in selected standard-risk patients who aim to achieve deeper remission. In patients with high-risk disease, maintenance with either bortezomib plus lenalidomide or daratumumab plus lenalidomide is recommended. Thalidomide maintenance may be used in patients who have received thalidomide-based induction therapy, continued until disease progression. However, the choice of maintenance agent, dose, and duration should be individualised on a case-by-case basis.	High	Round 1: 45% Round 2: 100%

DRd = daratumumab, lenalidomide, and dexamethasone; DVRd = daratumumab, bortezomib, lenalidomide, and dexamethasone; Isa-VRd = isatuximab, bortezomib, lenalidomide, and dexamethasone; MPT = melphalan, prednisone, and thalidomide; Rd = lenalidomide and dexamethasone; TIE = transplant-ineligible; VCd = bortezomib, cyclophosphamide, and dexamethasone; Vd = bortezomib and dexamethasone; VMP = bortezomib, melphalan, and prednisone; VRd = bortezomib, lenalidomide, and dexamethasone; VTd = bortezomib, thalidomide, and dexamethasone

**Table 7:** Consensus recommendations for treatment regimens for patients with renal dysfunction.

Statement	Recommendation	Grade of evidence	Consensus
31	Bortezomib- and daratumumab-based regimens can be used in patients with severe renal impairment, including those on dialysis, without the need for dose adjustment. Treatment can be modified to alternative regimens once renal function stabilises or improves.	Moderate	Round 1: 91%
32	For patients who are transplant-eligible with NDMM, add-on daratumumab significantly improves PFS, OS, CR, and MRD negativity compared to standard therapy alone and demonstrates similar efficacy in both patients with NDMM/RRMM, regardless of renal impairment status, making it a suitable recommendation for this population.	High	Round 1: 91%

CR = complete response; MRD = minimal residual disease; NDMM = newly diagnosed multiple myeloma; OS = overall survival; PFS = progression-free survival; RRMM = relapsed or refractory multiple myeloma.

**Table 8:** Consensus recommendations for RRMM patients.

Statement	Recommendation	Grade of evidence	Consensus
33	Regimens used after one to three prior therapies may be reused later in the disease course, particularly if relapse occurs at least six months after treatment discontinuation, with preference given to agents or classes that were not previously used or were used in only one prior line to maximise therapeutic benefit.	High	Round 1: 91%
34	Autologous HSCT is recommended for eligible patients without prior HSCT or for those with a sustained response following initial HSCT after the first relapse. Repeat autologous HSCT for relapsed conditions may be evaluated depending on the interval between the prior HSCT and documented progression. The outlook for patients who experience a relapse after autologous HSCT seems to vary based on how soon the relapse occurs. A remission period of at least two to three years is generally recommended before considering a second autologous HSCT.	High	Round 1: 73% Round 2: 91%
**At first relapse**			
35	In enhanced resource settings, early initiation of daratumumab is recommended for patients with RRMM to prevent poor outcomes associated with advanced disease characteristics^a^. This approach may improve response rates and overall prognosis.	High	Round 1: 91%
36	In patients who are lenalidomide-refractory, treatment options include DVd, DKd, Ixa-Pd, PVd, and KPd. For those who have received one prior line of therapy containing both lenalidomide and bortezomib, DPd or ixazomib or carfilzomib-containing regimens without lenalidomide are recommended. Thalidomide or cyclophosphamide-containing regimens can be used in resource-constrained settings.	High	Round 1: 64% Round 2: 91%
37	In patients who are bortezomib-refractory, treatment options include KRd, DKd, DRd, Isa-Kd, and KPd. Ixazomib-containing regimens may be considered in cases of logistical challenges or for frail patients. Thalidomide or cyclophosphamide-containing regimens can be used in resource-constrained settings (Grade of evidence: moderate).	High	Round 1: 64% Round 2: 100%
38	In patients who are anti-CD38 refractory, treatment options include KRd, KPd, and PVd.	High	Round 1: 82%
39	Patients with disease refractory to the novel agent in a doublet regimen should be considered for an alternative triplet therapy that excludes the drug they are no longer responding to.	High	Round 1: 100%
**After two or three lines of therapy**			
40	For second or subsequent relapses, consider drug classes not used during the first relapse. Regimens containing at least two agents to which the patient is not refractory from the first relapse options should be prioritised. Clinical trial enrolment is encouraged when feasible. Treatment options include Isa-Pd, DKd, DPd, Isa-Kd, Elo-Pd, and KPd. If daratumumab, carfilzomib, or elotuzumab is unavailable, PCd or Pd may be used. Bendamustine, thalidomide, cyclophosphamide or melphalan-containing regimens can be used for transplantin-eligible patients in resource-constrained settings (Grade of evidence: moderate).	High	Round 1: 82%
41	In patients who are anti-CD38 refractory and have received two prior lines of therapy, including lenalidomide and a PI, the treatment option is EPd. IxPd is recommended for those who have received two prior lines of therapy containing an IMiD and a PI, and with disease progression on/within 60 days of completion of the last therapy.	High	Round 1: 82%
42	In enhanced resource settings, CAR T-cell therapy can be an option for patients who are refractory after two prior lines of therapy that included an IMiD, an anti-CD38 mAb, and a PI.	High	Round 1: 73% Round 2: 91%
43	In patients with MM, SC daratumumab (1,800 mg) demonstrates a comparable safety profile to IV daratumumab, with shorter median administration time and reduced IRR rates. SC daratumumab is a viable and preferable alternative to IV daratumumab in eligible Asian patients. Also, the combination of daratumumab, thalidomide, and dexamethasone is effective in RRMM. The use of thalidomide and dexamethasone provides a cost-effective option that may improve access to daratumumab-based triplets.	High	Round 1: 82%
44	For RRMM patients, perform a thorough evaluation for transformation to aggressive disease such as plasma cell leukaemia or extramedullary disease, and consider repeat FISH to assess for t(11;14). For aggressive MM, regimens such as DCEP, DT-PACE, or VTD-PACE may be used. In cases with t(11;14), venetoclax/dexamethasone ± daratumumab or a PI may be considered.	High	Round 1: 91%

CAR T-cell = chimeric antigen receptor T-cell; CD38 = Cluster of differentiation 38; DCEP = dexamethasone, cyclophosphamide, etoposide, and cisplatin; DKd = daratumumab, carfilzomib, and dexamethasone; DPd = daratumumab, pomalidomide, and dexamethasone; DRd = daratumumab, lenalidomide, and dexamethasone; DT-PACE = daratumumab, thalidomide, dexamethasone, cisplatin, doxorubicin, cyclophosphamide, and etoposide; DVd = daratumumab, bortezomib, and dexamethasone; Elo-Pd = elotuzumab, pomalidomide, and dexamethasone; EPd = elotuzumab, pomalidomide, and dexamethasone; FISH = fluorescence in situ hybridisation; HSCT = haematopoietic stem cell transplantation; IMiD = immunomodulatory drug; IRR = infusion-related reaction; Isa-Kd = isatuximab, carfilzomib, and dexamethasone; Isa-Pd = isatuximab, pomalidomide, and dexamethasone; IV = intravenous; Ixa-Pd = ixazomib, pomalidomide, and dexamethasone; IxPd = ixazomib, pomalidomide, and dexamethasone; KPd = carfilzomib, pomalidomide, and dexamethasone; KRd = carfilzomib, lenalidomide, and dexamethasone; MM = multiple myeloma; PCd = pomalidomide, cyclophosphamide, and dexamethasone; PI = proteasome inhibitor; PVd = pomalidomide, bortezomib, and dexamethasone; RRMM = relapsed or refractory multiple myeloma; SC = subcutaneous; VTD-PACE = bortezomib, thalidomide, dexamethasone, cisplatin, doxorubicin, cyclophosphamide, and etoposide.

a Before patients become heavily pretreated or develop aggressive disease features such as mass lesions, including bone plasmacytomas or extramedullary plasmacytomas.

**Table 9:** Consensus recommendations for the management of late relapse (≥ 3 prior therapies).

Statement	Recommendation	Grade of evidence	Consensus
45	In enhanced resource settings, approved CAR T-cell therapy is recommended for patients who have relapsed after at least three prior lines of therapy.	High	Round 1: 55% Round 2: 91%
46	In limited resource settings, bendamustine, thalidomide, cyclophosphamide or melphalan-containing regimens can be used.	Moderate	Round 1: 91%
47	In enhanced resource settings, bispecific antibodies are recommended for patients who have received at least four prior therapies that included an anti-CD38 mAb, a PI, and an IMiD. When feasible, participation in a clinical trial is recommended. Available options in this setting include elranatamab-bcmm, talquetamab-tgvs, and teclistamab-cqyv.	High	Round 1: 73% Round 2: 100%
48	Selinexor in combination with dexamethasone may be considered for patients who have received at least four prior therapies and whose disease is refractory to at least two PIs, two IMiDs, and an anti-CD38 mAb.	High	Round 1: 82%
49	For patients who have received at least four prior therapies, including an anti-CD38 mAb, a PI, and an IMiD, belantamab mafodotin-blmf may be considered if available. Patients may be treated with more than one BCMA-targeted therapy^a^ for their disease. Although the optimal sequencing of BCMA-directed therapies is not yet established, emerging data suggest that immediate sequential use after relapse may result in lower response rates.	High	Round 1: 73% Round 2: 91%

BCMA = B-cell maturation antigen; CAR T-cell = chimeric antigen receptor T-cell; CD38 = Cluster of differentiation 38; IMiD = immunomodulatory drug; PI = proteasome inhibitor.

a BCMA-targeted therapies are linked to complications including ocular toxicities, CRS, ICANS, cytopenia, infections, HLH, and other systemic toxicities.

**Table 10:** Consensus recommendations for infection prevention in patients with MM.

Statement	Recommendation	Grade of evidence	Consensus
50	During periods of increased infectious risk^a^, antibacterial prophylaxis is recommended. Prophylactic antiviral treatment (Acyclovir) is also recommended for patients undergoing a regimen that includes a PI or CD38-targeted therapy.	High	Round 1: 91%
51	Patients with recurrent infections should undergo an evaluation of their immune status, including a detailed infection history and laboratory testing of immune parameters such as immunoglobulin levels, to determine eligibility for early IVIG therapy. Although the optimal IVIG dosing schedule is not clearly established, the treatment goal should be to prevent further infections.	High	Round 1: 91%
52	Hypogammaglobulinaemia is more common in patients treated with daratumumab. IVIG replacement should be considered based on individual risk, with IgG levels monitored to guide use. Primary IVIG supplementation has also been shown to reduce high-grade infections in patients receiving teclistamab. Further studies are needed to better identify patients at the highest infection risk and optimise IVIG use.	High	Round 1: 82%
53	Considering the enhanced safety profile of subcutaneously administered daratumumab, prolonged use of corticosteroids solely to prevent infusion-related reactions is not advised.	High	Round 1: 100%
54	Vaccinations play a crucial role in an effective infection prevention strategy for all patients. HCPs should consult the most up-to-date recommendations on seasonal vaccines and other vaccines relevant to adult patients. The timing of vaccinations should be considered based on the patient’s MM burden or disease response, as well as the time since receiving anti-myeloma treatments, especially high-dose chemotherapy or ASCT.	High	Round 1: 100%

ASCT = autologous stem cell transplant; CD38 = cluster of differentiation 38; HCPs = healthcare professionals; IgG = immunoglobulin G; IVIG = intravenous immunoglobulin; MM = multiple myeloma; PI = proteasome inhibitor.

a First three months after diagnosis and when treating RRMM.

**Table 11:** Consensus recommendation for high-risk SMM.

Statement	Recommendation	Grade of evidence	Consensus
55	Patients with high-risk SMM may benefit from early intervention, with single-agent lenalidomide as an option for carefully selected cases. Those with rising biomarkers or evolving risk features need close monitoring as low-risk disease can progress to high-risk over time, necessitating adjusted follow-up and management strategies. Daratumumab monotherapy can also be considered as an alternative.	High	Round 1: 82% Round 2: 100%

SMM = smouldering multiple myeloma

**Table 12:** Consensus recommendations for the management of bone disease in patients with MM.

Statement	Recommendation	Grade of evidence	Consensus
56	All patients receiving initial therapy for MM should receive bone-targeting agents, such as bisphosphonates^a^, for up to two years. Dosing frequency (monthly vs. every three months) should be individualised based on patient factors, treatment response, and the chosen agent. Extensions beyond two years should be guided by clinical judgement. Denosumab^b^ is an alternative option to bisphosphonates.	High	Round 1: 100%
57	Radiation therapy may be considered for patients with pain that does not respond to systemic treatment or in cases of bone lesions causing spinal cord compression. If the compression is due to retropulsed bone, surgery should be prioritised over radiation. Extensive use of radiotherapy, especially early in the disease for pain relief alone, should be avoided to preserve bone marrow function.	Moderate	Round 1: 100%
58	Orthopaedic consultation should be sought without delay for fractures, impending fractures, or instability in weight-bearing bones to consider surgical stabilisation. Postoperative radiation is rarely required in these cases, as systemic therapy typically provides adequate disease control.	Moderate	Round 1: 100%

MM = multiple myeloma;

a Monitor for renal dysfunction, and a baseline dental assessment is strongly recommended;

b Monitor for calcium level, and baseline dental assessment is recommended.
