# Supplementary material for: Malaysian Expert Consensus Guidelines for the Diagnosis and Management of Multiple Myeloma: A Modified Delphi Approach
Source: Malays J Med Sci. 2026 Aug 31;33(4):7–36. doi: 10.21315/mjms-03-2026-153 (PMC13706670; doi:10.21315/mjms-03-2026-153)
Supplement: Supplementary file 1 [file MJMS_33-4_2RA_Supplementary_file.pdf]

Supplementary

1. Search strategy:

("Multiple Myeloma"[Mesh] OR "multiple myeloma"[tiab] OR "plasma cell myeloma"[tiab]) AND ("Malaysia"[Mesh] OR Malaysia[tiab] OR Malaysian[tiab] OR Asia[tiab] OR Asian[tiab] OR "Asia"[Mesh]) AND (treatment[tiab] OR management[tiab] OR "Drug Therapy"[Mesh]) AND (symptomatic[tiab] OR asymptomatic[tiab] OR MGUS[tiab] OR SMM[tiab] OR "smouldering multiple myeloma"[tiab] OR "smoldering multiple myeloma"[tiab] OR diagnos*[tiab] OR prognos*[tiab] OR staging[tiab] OR "risk stratification"[tiab] OR ISS[tiab] OR "International Staging System"[tiab] OR R-ISS[tiab] OR "Revised International Staging System"[tiab] OR "response criteria"[tiab] OR MRD[tiab] OR "minimal residual disease"[tiab] OR NDMM[tiab] OR "newly diagnosed"[tiab] OR "treatment naive"[tiab] OR "transplant eligible"[tiab] OR ASCT[tiab] OR "autologous stem cell transplant"[tiab] OR "transplant ineligible"[tiab] OR frail[tiab] OR elderly[tiab] OR "renal impairment"[tiab] OR "renal insufficiency"[tiab] OR "kidney failure"[tiab] OR "kidney disease"[tiab] OR RRMM[tiab] OR relaps*[tiab] OR refractory[tiab] OR "early relapse"[tiab] OR "late relapse"[tiab] OR infection*[tiab] OR "infection prevention"[tiab] OR vaccination[tiab] OR vaccine*[tiab] OR ("high-risk"[tiab] AND (SMM[tiab] OR smoldering[tiab] OR smouldering[tiab])) OR "supportive care"[tiab] OR bisphosphonate*[tiab] OR "pain management"[tiab]) AND ("proteasome inhibitor*"[tiab] OR bortezomib[tiab] OR carfilzomib[tiab] OR ixazomib[tiab] OR "immunomodulatory drug*"[tiab] OR IMiD*[tiab] OR lenalidomide[tiab] OR thalidomide[tiab] OR pomalidomide[tiab] OR chemotherapy[tiab] OR "alkylating agent*"[tiab] OR cyclophosphamide[tiab] OR melphalan[tiab] OR daratumumab[tiab] OR isatuximab[tiab] OR elotuzumab[tiab] OR "anti-CD38"[tiab] OR "CD38 antibody"[tiab] OR "CAR-T"[tiab] OR "CAR T-cell"[tiab] OR "chimeric antigen receptor"[tiab] OR idecabtagene[tiab] OR ciltacabtagene[tiab] OR "bispecific antibody"[tiab] OR bispecific[tiab] OR teclistamab[tiab] OR talquetamab[tiab] OR consolidation[tiab] OR maintenance[tiab] OR dexamethasone[tiab] OR corticosteroid*[tiab])

Supplementary Table 1. Oxford Grading System

1A

Level of evidence	Therapy/prevention, aetiology/harm	Prognosis
**1a**	Systematic review (with homogeneity) of randomised controlled trials (RCTs)	Systematic review (with homogeneity) of inception cohort studies; clinical decision rule validated in different populations
**1b**	Individual RCT [with narrow confidence interval (CI)]	Individual inception cohort study with >80% follow-up; clinical decision rule validated in a single population
**1c**	All or none	All or none case series
**2a**	Systematic review (with homogeneity) of cohort studies	Systematic review (with homogeneity) of either retrospective cohort studies or untreated control groups in RCTs
**2b**	Individual cohort study (including low-quality RCTs; e.g. <80% follow-up)	Retrospective cohort study or follow-up of untreated control patients in an RCT; derivation of a clinical decision rule or validated on a split-sample only
**2c**	‘Outcomes’ research and ecological studies	‘Outcomes’ research
**3a**	Systematic review (with homogeneity) of case–control studies	
**3b**	Individual case–control study	
**4**	Case series (and poor-quality cohort and case–control studies)	Case series (and poor-quality prognostic cohort studies)
**5**	Expert opinion without explicit critical appraisal, or based on physiology, bench research, or ‘first principles’	Expert opinion without explicit critical appraisal, or based on physiology, bench research, or ‘first principles’

1B

Code	Quality of evidence	Definition
**A**	**High**	Further research is very unlikely to change our confidence in the estimate of effect.<br>• Several high-quality studies with consistent results<br>• In special cases: one large, high-quality multi-centre trial
**B**	**Moderate**	Further research is likely to have an important impact on our confidence in the estimate of effect and may cha nge the estimate.<br>• One high-quality study<br>• Several studies with some limitations
**C**	**Low**	Further research is very likely to have an important impact on our confidence in the estimate of effect and is li kely to change the estimate.<br>• One or more studies with severe limitations
**D**	**Very low**	Any estimate of the effect is very uncertain.<br>• Expert opinion<br>• No direct research evidence<br>• One or more studies with very severe limitations

Supplementary Table 2: Recommendations with level of evidence

S. No.	Statements	Evidence	Study type	Level of evidence	Grade of evidence	Round 1 voting consensus (%)	Round 2 voting consensus (%)
1	The diagnosis of symptomatic MM requires:<br>➢ Clonal BMPCs ≥10% or a biopsy-proven plasmacytoma, and evidence of any one or more of the following MDEs:<br>• Increased calcium levels (>2.75 mmol/L)<br>• Renal insufficiency (creatinine >177 µmol/L or creatinine clearance <40 mL/min)<br>• Anaemia (haemoglobin <10 g/dL or ≥2 g/dL below the	NCCN Clinical Practice Guidelines in Oncology (Multiple Myeloma) (NCCN 2025)<br><br>Multiple myeloma: 2024 update on diagnosis, risk stratification, and management (Rajkumar 2024)<br><br>Singapore Myeloma Study Group consensus guidelines for the management of patients with newly diagnosed multiple myeloma (de Mel et al. 2025)	CPG<br><br>IMWG guidelines<br><br>Consensus guidelines	1a<br><br>1a<br><br>5	High	82%	100%

	lower limit of normal)<br>• ≥1 osteolytic bone lesions identified through skeletal radiography, CT, or FDG-PET/CT<br>➢ Additional MDEs include (SLiM features):<br>• Clonal BMPCs ≥60%<br>• sFLCr of ≥100 with an involved FLC concentration of at least 100 mg/L<br>• More than one focal lesion ≥5 mm detected on MRI.						
2	It is characterised by the presence of serum monoclonal protein levels of ≥30 g/L or Bence Jones protein levels of ≥500 mg/24 hours, and/or clonal	NCCN Clinical Practice Guidelines in Oncology (Multiple Myeloma) (NCCN 2025)<br><br>IMWG updated criteria for the diagnosis of multiple myeloma (Rajkumar et al. 2014)	CPG<br><br>IMWG guidelines	1a<br><br>1a	High	64%	100%

	BMPCs comprising 10%–60% of the marrow. SMM is distinguished from symptomatic MM by the absence of MDEs or amyloidosis.	Multiple myeloma: 2024 update on diagnosis, risk stratification, and management (Rajkumar 2024)	IMWG guidelines	1a			
		Patterns of progression in a contemporary cohort of 447 patients with SMM (Werly et al. 2024)	Retrospective study	2b			
		Risk and patterns of progression among SMM patie nts after the implementation of new criteria and modern imagin g (Kastritis et al. 2024**)**	Retrospective study	2b			
3	At diagnosis, BMPCs >20%, M protein >20 g/L, and sFLCr >20 are key variables for risk stratification. Patients presenting with ≥2 of these risk factors are considered to have a high risk of 50%–80% progression to symptomatic MM within 2 years from diagnosis.	NCCN Clinical Practice Guidelines in Oncology (Multiple Myeloma) (NCCN 2025)	CPG	1a	High	82%	NA
		IMWG risk stratification model for SMM (Mateos et al. 2020)	IMWG guidelines	1a			
		Multiple myeloma: 2024 update on diagnosis, risk stratification, and management (Rajkumar 2024)	IMWG guidelines	1a			
		Risk stratification of SMM incorporating revised IMWG	Retrospective study	2b			

		diagnostic criteria (Lakshman et al. 2018)<br><br>The changing landscape of SMM: A European perspective (Caers et al. 2016)	 Expert recommendations	 5			
4	Diagnosis of MM should include testing for M proteins using a combination of SPEP, SIFE, and the sFLC assay when the disease is clinically suspected. At the time of initial diagnosis, bone marrow studies should include chromosome analysis using FISH performed on plasma cells obtained from bone marrow aspiration. The FISH panel, used for prognostic assessment, should evaluate for abnormalities such as del(13), del(17p13), t(4;14), t(11;14), t(14;16), t(14;20), 1q21 gain/amplification, and 1p deletion. This information helps define	NCCN Clinical Practice Guidelines in Oncology (Multiple Myeloma) (NCCN 2025)<br><br>Multiple myeloma: 2024 update on diagnosis, risk stratification, and management (Rajkumar 2024)<br><br>Singapore Myeloma Study Group consensus guidelines for the management of patients with newly diagnosed multiple myeloma (de Mel et al. 2025)	CPG<br><br>IMWG guidelines<br><br>Consensus guidelines	1a<br><br>1a<br><br>5	High	100%	NA

	the biological subtype of the disease, guides prognostic evaluation, and supports the determination of eligibility for clinical trials.						
5	To evaluate treatment response, M protein levels should be monitored 1-3 months during therapy using SPEP and sFLC assays, and every 3–4 months once therapy has ended. The sFLC assay is valuable, especially for patients without a measurable M protein when the FLCr is abnormal, or the involved FLC level is ≥100 mg/L. Additionally, urine protein electrophoresis may be performed every 3–6 months to monitor urinary M protein levels.	NCCN Clinical Practice Guidelines in Oncology (Multiple Myeloma) (NCCN 2025)<br><br>Multiple myeloma: 2024 update on diagnosis, risk stratification, and management (Rajkumar 2024)	CPG<br><br>IMWG guidelines	1a<br><br>1a	High	45%	91%
6	Diagnosis of bone involvement in MM is best achieved using low-dose WB-CT or PET/CT,	NCCN Clinical Practice Guidelines in Oncology (Multiple Myeloma) (NCCN 2025)	CPG	1a	High	82%	NA

	which offers superior sensitivity compared to CSS. MRI is particularly valuable for evaluating patients with suspected SMM as it identifies	Multiple myeloma: 2024 update on diagnosis, risk stratification, and management (Rajkumar 2024)	IMWG guidelines	1a			
	focal marrow lesions before osteolytic alterations, evaluates extramedullary disease, assesses spinal cord compression, and provides detailed imaging of symptomatic regions. CSS should be reserved for scenarios where advanced imaging options are not accessible.	Singapore Myeloma Study Group consensus guidelines for the management of patients with newly diagnosed multiple myeloma (de Mel et al. 2025)	Consensus guidelines	5			
7	In enhanced resource settings, NGS of bone marrow can be performed to identify	NCCN Clinical Practice Guidelines in Oncology (Multiple Myeloma) (NCCN 2025)	CPG	1a	Moderate	36%	100%
	significant genetic abnormalities, providing crucial insights for improved risk stratification. While NGS	Multiple myeloma: EHA-ESMO Clinical Practice Guidelines for diagnosis, treatment and follow-up (Dimopoulos et al. 2021)	CPG	1a			
	is not mandatory, it is beneficial when available and can enhance	NGS-Based Molecular Karyotyping of Multiple Myeloma: Results from the	Clinical trial	2b			

	treatment decision-making.	GEM12 Clinical Trial (Rosa-Rosa et al. 2022)					
8	Those with active MM can be staged using ISS. The ISS identifies three stages based on serum β-2 microglobulin and serum albumin levels. The R-ISS further incorporates prognostic information from LDH levels and high-risk chromosomal abnormalities, including t(4;14), t(14;16), and 17p13 deletion, as detected by FISH. Other staging systems, such as MASS or mSmart, can also be considered (Grade of evidence: moderate).	NCCN Clinical Practice Guidelines in Oncology (Multiple Myeloma) (NCCN 2025)<br><br>Revised International Staging System for multiple myeloma: A report from IMWG (Palumbo et al. 2015)<br><br>Singapore Myeloma Study Group consensus guidelines for the management of patients with newly diagnosed multiple myeloma (de Mel et al. 2025)<br><br>Prognostic evaluation and staging optimisation of the MASS in the real world for newly diagnosed multiple myeloma patients (Cao et al. 2023)<br><br>Performance of newer staging systems for myeloma in a contemporary, large cohort of patients in the United States (Mohyuddin et al. 2023)	CPG<br><br>IMWG guidelines<br><br>Consensus guidelines<br><br>Retrospective study<br><br>Retrospective study	1a<br><br>1a<br><br>5<br><br>2b<br><br>2b	High	100%	NA
9	High-risk NDMM is defined by the presence	International Myeloma Society/IMWG consensus	IMWG guidelines	1a	High	73%	100%

of at least one of the following abnormalities:<br>• del(17p), with a cutoff of >20% clonal fraction, and/or TP53 mutation<br>• An IgH translocation including t(4;14), t(14;16), or t(14;20) along with 1q+ and/or del(1p32)<br>• Monoallelic del(1p32) along with 1q+ or biallelic del(1p32)<br>• β2 microglobulin ≥5.5 mg/L with normal creatinine (<106 µmol/L).<br><br>Patients presenting with ≥2 high-risk cytogenetic abnormalities are considered to have a very high risk of disease progression or relapse.	recommendations on the definition of high-risk multiple myeloma (Avet-Loiseau et al. 2025)		
	Singapore Myeloma Study Group consensus guidelines for the management of patients with newly diagnosed multiple myeloma (de Mel et al. 2025)	Consensus guidelines	5
	Diagnosis and initial treatment of transplant-eligible high-risk myeloma patients: A British Society for Haematology/UK Myeloma Society Good Practice Paper (Kaiser et al. 2024)	Recommendations	5

10	Patients with relapsed MM are considered at high risk of further progression or relapse if they meet any of the following criteria:<br>• Disease relapse within 2 years of initial therapy when transplant and maintenance were used<br>• Disease relapse within 18 months when treated with nontransplant-based therapy<br>• Acquisition of high-risk cytogenetic abnormalities, such as 1q gain/1q21 amplification and/or del(17p)/TP53 mutation<br>• Presence of extramedullary disease at relapse	NCCN Clinical Practice Guidelines in Oncology (Multiple Myeloma) (NCCN 2025)	CPG	1a	High	100%	NA

	and/or circulating plasma cells						
11	As per IMWG response criteria (2016), MM is categorised into four levels: sCR, CR, VGPR, and PR, which helps assess and classify responses to treatment in clinical trials and routine practice, helping to ensure consistent reporting and comparison of treatment efficacy. Additional categories, such as MR, PD, and SD, are also crucial for accurate assessment of treatment outcomes.	IMWG consensus criteria for response and minimal residual disease assessment in multiple myeloma (Kumar et al. 2016)<br><br>Utility of repeating bone marrow biopsy for confirmation of CR in multiple myeloma (Tschautscher et al. 2020)	IMWG guidelines<br><br>Retrospective study	1a<br><br>2b	High	91%	NA
12	The MM response criteria are recommended for assessing disease status in patients with MM. If some, but not all, criteria are met at any response level, the disease status should be downgraded to the next lower level of response category.	Multiple myeloma: 2024 update on diagnosis, risk stratification, and management (Rajkumar 2024)	IMWG guidelines	1a	High	100%	NA

13	In enhanced resource settings, when MRD assessment is available, sustained MRD-negative status may be verified by confirming bone marrow negativity using NGF or NGS techniques with a minimum sensitivity of 1 in 10^5 nucleated cells or higher, with repeat evaluations performed at least 1 year apart.	NCCN Clinical Practice Guidelines in Oncology (Multiple Myeloma) (NCCN 2025)<br><br>IMWG consensus criteria for response and minimal residual disease assessment in multiple myeloma (Kumar et al. 2016)<br><br>Association of minimal residual disease with superior survival outcomes in patients with multiple myeloma: A meta-analysis (Munshi et al. 2017)	CPG<br><br>IMWG guidelines<br><br>Meta-analysis	1a<br><br>1a<br><br>1a	High	45%	91%
14	The first step in treating patients with NDMM is to assess their eligibility for high-dose therapy and ASCT, taking into account factors such as age and comorbidities. Advanced age and renal dysfunction, however, are not absolute contraindications to transplant. Therefore, early referral to an HCT centre for evaluation of transplant eligibility is recommended if the	NCCN Clinical Practice Guidelines in Oncology (Multiple Myeloma) (NCCN 2025)<br>Multiple myeloma: 2022 update on diagnosis, risk stratification an d Management (Rajkumar 2022)<br><br>Multiple myeloma: 2024 update on diagnosis, risk stratification, and management (Rajkumar 2024)<br><br>Evolving strategies in the management of transplant-eligible patients with newly diagnosed multiple myeloma (Perrot 2025)	CPG<br><br>IMWG guidelines<br><br>IMWG guidelines<br><br>Review	1a<br><br>1a<br><br>1a<br><br>5	High	82%	NA

	patient is willing to undergo ASCT.						
15	Three drug regimens should be the preferred minimum induction option for transplant-eligible patients. These triplet regimens should include two different drug classes (PI and IMiD) and a corticosteroid. In enhanced resource settings, where available, a quadruplet regimen can be considered as initial therapy based on improved RR, DpR, and PFS. Quadruplet regimens should include an anti-CD38 mAb, two different drug classes (PI and IMiD), and a corticosteroid. Frailty assessment should be performed in older adults, with consideration of dose modifications, particularly steroids, based on functional status and age.	NCCN Clinical Practice Guidelines in Oncology (Multiple Myeloma) (NCCN 2025)	CPG	1a	High	73%	91%
		Multiple myeloma: 2024 update on diagnosis, risk stratification, and management (Rajkumar 2024)	IMWG guidelines	1a			
		A systematic review on the epidemiology and treatment options of multiple myeloma in Asia (Chng et al. 2024)	SLR	1a			
		Quadruplet regimens for patients with newly diagnosed multiple myeloma: a systematic review and meta-analysis (Ebraheem et al. 2024)	SLR	1a			
		Daratumumab-based quadruplet versus triplet induction regimens in transplant-eligible newly diagnosed multiple myeloma: a systematic review and meta-analysis (Souto Filho et al. 2025)	SLR	1a			

16	Doublet regimens are not recommended for transplant-eligible candidates; they are reserved for patients who are TIE with poor performance or frail status. A third drug may be added once the patient's performance status improves.	NCCN Clinical Practice Guidelines in Oncology (Multiple Myeloma) (NCCN 2025)	CPG	1a	High	100%	NA
17	First-line three drug regimens options for transplant-eligible patients are VRd, VTd, VCd, and KRd.	NCCN Clinical Practice Guidelines in Oncology (Multiple Myeloma) (NCCN 2025)	CPG	1a	High	73%	100%
		Multiple myeloma: 2024 update on diagnosis, risk stratification, and management (Rajkumar 2024)	IMWG guidelines	1a			
		The Singapore Myeloma Study Group Consensus Guidelines for the management of patients with multiple Myeloma (de Mel et al. 2017)	Consensus guidelines	5			
		A systematic review on the epidemiology and treatment options of Multiple Myeloma in Asia (Chng et al. 2024)	SLR	1a			

		Bortezomib, thalidomide, and dexamethasone versus bortezomib, lenalidomide, and dexamethasone in transplant-eligible newly diagnosed multiple myeloma: a systemic review and meta-analysis (Wang et al. 2025)	SLR	1a			
		The establishment of multiple myeloma Clinical registry in the Asia–Pacific region: The Asia–Pacific Myeloma and Related Diseases Registry (APAC MRDR) (Aoki et al. 2024)	Retrospective study	2b			
		Treatment pattern and outcome of newly diagnosed multiple myeloma patients in a resource-limited setting (Cheong et al. 2024)	Retrospective study	2b			
18	In enhanced resource settings, first-line anti-CD38-containing regimens for transplant-eligible patients are DVRd, DVTd or Isa-VRd. Induction with 3 to 4 cycles of the regimen is recommended.	NCCN Clinical Practice Guidelines in Oncology (Multiple Myeloma) (NCCN 2025)	CPG	1a	High	64%	100%
		Multiple myeloma: 2024 update on diagnosis, risk stratification, and management (Rajkumar 2024)	IMWG guidelines	1a			

		Daratumumab-based induction therapy for multiple myeloma: A systematic review and meta-analysis (Chong et al. 2021)	SLR	1a			
		Treatment of newly diagnosed myeloma (mSMART 2024)	mSMART guidelines	1a			
		Expert consensus on the incorporation of anti-CD38 mAb therapy into the mana gement of newly diagnosed multiple myel oma (Lonial et al. 2023)	Consensus guidelines	5			
		Bortezomib, thalidomide, and dexamethasone with or without daratumumab for transplantation-eligible patients with newly diagnosed multiple myeloma (CASSIOPEIA): health-related quality of life outcomes of a randomised, open-label, phase 3 trial (Roussel et al. 2020)	RCT	1b			
19	VTD-PACE may be considered for patients who are transplant-eligible with aggressive MM, such as those with extramedullary disease or plasma cell leukaemia.	NCCN Clinical Practice Guidelines in Oncology (Multiple Myeloma) (NCCN 2025)	CPG	1a	High	82%	NA
		The efficacy of new drug regimens in treating newly diagnosed high-risk cytogenetic	SLR	1a			

	Other treatment options for high-risk MM in transplant-eligible patients include DCVRd for ultra-high-risk MM (i.e. ≥2 high-risk cytogenetic aberrations or plasma cell leukaemia). Additional options include DKRd, Isa-KRd, and KRd with or without tandem autologous or allogeneic HSCT, particularly for plasma cell leukaemia.	multiple myeloma patients: a systematic literature review and meta-analysis (Zhou et al. 2025)					
		VTd-PACE and VTd-PACE-like regimens are effective salvage therapies in difficult-to-treat relapsed/refractory multiple myeloma: a single-centre experience (Ghandili et al. 2022)	Retrospective study	2b			
		Diagnosis and initial treatment of transplant-eligible high-risk myeloma patients: A British Society for Haematology/UK Myeloma Society Good Practice Paper (Kaiser et al. 2024)	Recommendations	5			
		Daratumumab, cyclophosphamide, bortezomib, lenalidomide, and dexamethasone as induction and extended consolidation improves outcome in ultra-high-risk multiple myeloma (Kaiser et al. 2023)	RCT	1b			
20	Early ASCT is recommended for eligible NDMM patients following 3–6 cycles of induction therapy; selected high-risk	NCCN Clinical Practice Guidelines in Oncology (Multiple Myeloma) (NCCN 2025)	CPG	1a	High	91%	NA
		Multiple myeloma: 2024 update on diagnosis, risk stratification,	IMWG guidelines	1a			

	patients, especially those not achieving CR after their initial transplant, may be considered for additional consolidation cycles, especially in patients with high-risk cytogenetic abnormalities and ultra-high-risk MM.	and management (Rajkumar 2024)					
		ASTCT clinical practice recommendations for transplantation and cellular therapies in multiple myeloma (Dhakal et al. 2022)	CPG	1a			
		Autologous haematopoietic stem-cell transplantation versus bortezomib–melphalan–prednisone, with or without bortezomib–lenalidomide–dexamethasone consolidation therapy, and lenalidomide maintenance for newly diagnosed multiple myeloma (EMN02/HO95): a multicentre, randomised, open-label, phase 3 study	RCT	1b			
21	Consolidation with CAR T-cell therapy is not widely recommended following first-line therapy outside of clinical trial settings.	ASTCT clinical practice recommendations for transplantation and cellular therapies in multiple myeloma (Dhakal et al. 2022)	CPG	1a	High	100%	NA
22	Maintenance therapy with lenalidomide is recommended for a duration of 2 to 4 years as the standard approach,	NCCN Clinical Practice Guidelines in Oncology (Multiple Myeloma) (NCCN 2025)	CPG	1a	High	73%	100%

with consideration of longer treatment on a case-by-case basis depending on tolerability and toxicities.	Multiple myeloma: 2024 update on diagnosis, risk stratification, and management (Rajkumar 2024)	IMWG guidelines	1a
Thalidomide may be considered as an alternative depending on tolerability, access, and concerns regarding second malignancies associated with lenalidomide.	Treatment of newly diagnosed myeloma (mSMART 2024)	mSMART guidelines	1a
	Singapore Myeloma Study Group consensus guidelines for the management of patients with newly diagnosed multiple myeloma (de Mel et al. 2025)	Consensus guidelines	5
	maintenance treatment and survival in patients with myeloma: A systematic review and network meta-analysis (Gay et al. 2018)	SLR	1a
	Defining the optimal duration of lenalidomide maintenance after autologous stem cell transplant - Data from the Myeloma XI Trial (Pawlyn et al. 2022)	RCT	1b
	Thalidomide maintenance therapy for patients with multiple myeloma: meta-analysis (Kagoya and Kurokawa, 2012)	Meta-analysis	1a

23	Two-drug maintenance therapy can be considered for high-risk MM, and the options can be bortezomib plus lenalidomide or daratumumab plus lenalidomide.	NCCN Clinical Practice Guidelines in Oncology (Multiple Myeloma) (NCCN 2025)	CPG	1a	High	64%	91%
		Multiple myeloma: 2024 update on diagnosis, risk stratification, and management (Rajkumar 2024)	IMWG guidelines	1a			
		Singapore Myeloma Study Group consensus guidelines for the management of patients with newly diagnosed multiple myeloma (de Mel et al. 2025)	Consensus guidelines	5			
		Daratumumab with lenalidomide as maintenance after transplant in newly diagnosed multiple myeloma: The AURIGA study (Badros et al. 2025)	RCT	1b			
		Maintenance therapy for cytogenetically high-risk multiple myeloma: landscape in the era of novel drugs (Gu et al. 2024)	Meta-analysis	1a			
24	Thalidomide maintenance therapy may be a possible approach for standard-risk patients, showing significantly prolonged median PFS compared to no	Maintenance treatment and survival in patients with myeloma: A systematic review and network meta-analysis (Gay et al. 2018)	SLR	1a	High	73%	91%

	maintenance therapy. Its primary adverse effect is peripheral neuropathy, but advantages include the low risk of neutropenia, minimal adverse impact on stem cell harvest, and no requirement for dose adjustments in patients with renal impairment. However, thalidomide maintenance is associated with inferior PFS and OS in patients with unfavourable cytogenetics or high-risk MM.	The role of maintenance thalidomide therapy in multiple myeloma: MRC Myeloma IX results and meta-analysis (Morgan et al. 2012)<br><br>Thalidomide maintenance therapy for patients with multiple myeloma: meta-analysis (Kagoya and Kurokawa, 2012)	RCT<br><br>Meta-analysis	1b<br><br>1a			
25	Stem cell toxins, such as nitrosoureas or alkylating agents, can reduce stem cell reserves. Regimens containing these agents, particularly melphalan, should be avoided in patients who may be candidates for HSCT until stem cells are harvested. If HSCT is delayed, stem cells should be collected and stored. It is	NCCN Clinical Practice Guidelines in Oncology (Multiple Myeloma) (NCCN 2025)<br><br>Multiple myeloma: 2024 update on diagnosis, risk stratification, and management (Rajkumar 2024)<br><br>Impact of interval progression before autologous stem cell transplant in patients with multiple myeloma (Bao et al. 2023)	CPG<br><br>IMWG guidelines<br><br>Retrospective study	1a<br><br>1a<br><br>2b	Moderate	100%	NA

	recommended to harvest peripheral blood stem cells after 4–6 cycles of therapy, before prolonged exposure to lenalidomide and/or daratumumab, if HSCT is being considered.						
26	Stem cell mobilisation should be performed with G-CSF, with or without prior chemotherapy using cyclophosphamide at a dose range of 1.5–4 g/m². Additionally, plerixafor, a selective and reversible CXCR4 antagonist, should be considered to decrease mobilisation failure by inhibiting HSC adhesion to the marrow and promoting their circulation.	Effectiveness and safety of low-dose etoposide chemomobilisation in patients with multiple myeloma and lymphoma (Mercan Sarıdaş, Ozkocaman, and Özkalemkaş 2025)<br><br>Stem cell mobilisation in multiple myeloma: Comparing safety and efficacy of cyclophosphamide +/− plerixafor versus granulocyte colony-stimulating factor +/− plerixafor in the lenalidomide era (Johnsrud et al. 2022)<br><br>Multiple myeloma: The role of ASCT in the era of immunotherapy (Rocchi et al. 2024)	Retrospective study<br><br>Retrospective study<br><br>Review	2b<br><br>2b<br><br>5	Moderate	82%	NA
27	Stem cell mobilisation may be reduced after induction with lenalidomide- or	Stem cell mobilisation yields with daratumumab- and lenalidomide-containing quadruplet induction	RCTs	1a	High	64%	100%

daratumumab-containing regimens. Early stem cell mobilisation after 2 to 4 cycles of induction therapy is encouraged. An upfront plerixafor strategy may be considered in patients at high risk of poor mobilisation, particularly in enhanced resource centres.	therapy in newly diagnosed multiple myeloma: Findings from the MASTER and GRIFFIN trials (Chhabra et al. 2023)		
	Impact of anti-CD38 mAb therapy on CD34+ hematopoietic stem cell mobilisation, collection, and engraftment in multiple myeloma patients—A systematic review (Bigi et al. 2024)	SLR	1a
	Stem cell mobilisation yields with daratumumab (dara) and lenalidomide (len)-containing quadruplet induction therapy in patients with newly diagnosed multiple myeloma (NDMM): A real-world experience at two institutes (Varga et al. 2024)	Retrospective study	2b
	Multiple myeloma: The role of ASCT in the era of immunotherapy (Rocchi et al. 2024)	Review	5

28	For TIE patients, first-line therapy should be individualised based on frailty, myeloma risk profile, and financial affordability. Based on published evidence, VRd or DRd is recommended as first-line therapy, while DVRd or Isa VRd may offer an advantage in fitter patients. Rd or Vd can be considered for selected very frail patients. Induction therapy should be continued for 8 to 12 cycles or until response plateaus.	NCCN Clinical Practice Guidelines in Oncology (Multiple Myeloma) (NCCN 2025)	CPG	1a	High	73%	91%
		Multiple myeloma: 2024 update on diagnosis, risk stratification, and management (Rajkumar 2024)	IMWG guidelines	1a			
		Treatment of newly diagnosed myeloma (mSMART 2024)	mSMART guidelines	1a			
		Treatment regimens for transplant-ineligible patients with newly diagnosed multiple myeloma: A systematic literature review and network meta-analysis (Facon et al. 2022)	SLR	1a			
		A systematic literature review and network meta-analysis of treatments for patients with untreated multiple myeloma not eligible for stem cell transplantation (Weisel et al. 2016)	SLR	1a			
29	For TIE patients with limited resources, the combinations of VTd, VCd, VMP, and MPT	Recent advances in the management of multiple myeloma: clinical impact based on resource-stratification.	AMN guidelines	1a	High	64%	100%

can be used as an alternative induction therapy. The respective regimen can be dose-adjusted for frail patients.	Consensus statement of the Asian Myeloma Network at the 16th International Myeloma Workshop (Tan et al. 2018)		
	Phase II clinical trial of personalised VCD-VTD sequential therapy using the Vulnerable Elders Survey-13 (VES-13) for transplant-ineligible patients with newly diagnosed multiple myeloma (Nakazato et al. 2021)	RCT	1b
	The cost-effectiveness analysis of transplant-ineligible myeloma patients with Bortezomib plus Thalidomide plus Dexamethasone (VTD) or Bortezomib plus Melphalan plus Prednisolone (VMP) treatment in Southern Taiwan (Du et al. 2022)	Retrospective study	2b
	Frontline treatment of elderly non–transplant-eligible multiple myeloma patients using CyBorD with or without thalidomide-based consolidation: a retrospective multi-centre analysis of real-world data (Chan et al. 2019)	Retrospective study	2b

30	In TIE patients, those who have received VRd, DRd, or DVRd as induction therapy should be followed by lenalidomide maintenance. Daratumumab and lenalidomide maintenance can be considered in selected standard-risk patients who aim to achieve deeper remission. In patients with high-risk disease, maintenance with either bortezomib plus lenalidomide or daratumumab plus lenalidomide is recommended. Thalidomide maintenance may be used in patients who have received thalidomide-based induction therapy, continued until disease progression. However, the choice of maintenance agent, dose,	Multiple myeloma: 2024 update on diagnosis, risk stratification, and management (Rajkumar 2024)	IMWG guidelines	1a	High	45%	100%
		Treatment of newly diagnosed myeloma (mSMART 2024)	mSMART guidelines	1a			
		Singapore Myeloma Study Group consensus guidelines for the management of patients with newly diagnosed multiple myeloma (de Mel et al. 2025)	Consensus guidelines	5			

	and duration should be individualised on a case-by-case basis.						
31	Bortezomib- and daratumumab-based regimens can be used in patients with severe renal impairment, including those on dialysis, without the need for dose adjustment. Treatment can be modified to alternative regimens once renal function stabilises or improves.	NCCN Clinical Practice Guidelines in Oncology (Multiple Myeloma) (NCCN 2025)<br><br>Daratumumab, bortezomib, and dexamethasone for treatment of patients with relapsed or refractory multiple myeloma and severe renal impairment: Results from the Phase 2 GMMG-DANTE Trial (Leypoldt et al. 2023)<br><br>Safety and efficacy of new drug combinations in newly diagnosed multiple myel oma with renal failure at onset: A retrospective multicentric study (Frolli et al. 2023)	CPG<br><br>RCT<br><br>Retrospective study	1a<br><br>1b<br><br>2b	Moderate	91%	NA
32	For patients who are transplant-eligible with NDMM, add-on daratumumab significantly improves PFS, OS, CR, and MRD negativity compared to standard therapy alone	Efficacy of daratumumab on multiple myeloma patients with renal insufficiency: A systematic review and meta-analysis (Jiang et al. 2024)	SLR	1a	High	91%	NA

	and demonstrates similar efficacy in both patients with NDMM/RRMM, regardless of renal impairment status, making it a suitable recommendation for this population.						
33	Regimens used after 1–3 prior therapies may be reused later in the disease course, particularly if relapse occurs at least 6 months after treatment discontinuation, with preference given to agents or classes that were not previously used or were used in only one prior line to maximise therapeutic benefit.	NCCN Clinical Practice Guidelines in Oncology (Multiple Myeloma) (NCCN 2025)<br><br>Multiple myeloma: 2024 update on diagnosis, risk stratification, and management (Rajkumar 2024)	CPG<br><br>IMWG guidelines	1a<br><br>1a	High	91%	NA
34	Autologous HSCT is recommended for eligible patients without prior HSCT or for those with a sustained response following initial HSCT after the first relapse. Repeat autologous HSCT for relapsed conditions may be evaluated depending on the interval	NCCN Clinical Practice Guidelines in Oncology (Multiple Myeloma) (NCCN 2025)<br><br>Treatment of relapsed and refractory multiple myeloma: Recommendations from the IMWG (Moreau et al. 2021)<br><br>Salvage ASCT for multiple myeloma relapsing or progressing	CPG<br><br>IMWG guidelines<br><br>Retrospective study	1a<br><br>1a<br><br>2b	High	73%	91%

	between the prior HSCT and documented progression. The outlook for patients who experience a relapse after autologous HSCT seems to vary based on how soon the relapse occurs. A remission period of at least 2–3 years is generally recommended before considering a second autologous HSCT.	after upfront autologous transplantation (Auner et al. 2013)					
		Autologous retransplantation for patients with recurrent multiple myeloma: A single-centre experience with 200 patients (Sellner et al. 2013)	Retrospective study	2b			
35	In enhanced-resource settings, early initiation of daratumumab is recommended for patients with RRMM to prevent poor outcomes associated with advanced disease characteristics. This approach may improve response rates and overall prognosis.	NCCN Clinical Practice Guidelines in Oncology (Multiple Myeloma) (NCCN 2025)	CPG	1a	High	91%	NA
		Efficacy and safety of daratumumab in the treatment of relapsed/refractory multiple myeloma: A meta-analysis of randomised controlled trials (Huang et al. 2023)	SLR	1a			
		Daratumumab therapy for relapsed or refractory multiple myeloma: A single-centre retrospective study (Sunami et al. 2020)	Retrospective study	2b			

36	In patients who are lenalidomide-refractory, treatment options include DVd, DKd, Ixa-Pd, PVd, and KPd. For those who have received one prior line of therapy containing both lenalidomide and bortezomib, DPd or ixazomib or carfilzomib-containing regimens without lenalidomide are recommended. Thalidomide or cyclophosphamide-containing regimens can be used in resource-constrained settings.	NCCN Clinical Practice Guidelines in Oncology (Multiple Myeloma) (NCCN 2025)	CPG	1a	High	64%	91%
		Alliance A061202: Ixazomib, pomalidomide, and dexamethasone for patients with lenalidomide-refractory MM in first relapse (Voorhees et al. 2024)	RCT	1b			
		OS with daratumumab, bortezomib, and dexamethasone in previously treated multiple myeloma (CASTOR): A randomised, open-label, Phase III Trial (Sonneveld et al. 2023)	RCT	1b			
		Efficacy of thalidomide-based therapy following lenalidomide plus dexamethasone in patients with relapsed/refractory multiple myeloma (Kukreti et al. 2013)	Retrospective study	2b			
		Pomalidomide, bortezomib, and dexamethasone for patients with relapsed or refractory multiple myeloma previously treated with lenalidomide (OPTIMISMM): a randomised, open-label, phase 3 trial (Richardson et al. 2019)	RCT	1b			

37	In patients who are bortezomib-refractory, treatment options include KRd, DKd, DRd, Isa-Kd, and KPd. Ixazomib-containing regimens may be considered in cases of logistical challenges or for frail patients. Thalidomide or cyclophosphamide-containing regimens can be used in resource-constrained settings (Grade of evidence: moderate).	NCCN Clinical Practice Guidelines in Oncology (Multiple Myeloma) (NCCN 2025)	CPG	1a	High	64%	100%
		Efficacy of thalidomide-based therapy following lenalidomide plus dexamethasone in patients with relapsed/refractory multiple myeloma (Kukreti et al. 2013)	Retrospective study	2b			
		Comparative efficacy of treatments for previously treated multiple myeloma: a systematic literature review and network meta-analysis (Maiese et al. 2018)	SLR	1a			
		Carfilzomib, lenalidomide, and dexamethasone for relapsed multiple myeloma (Stewart et al. 2015)	RCT	1b			
		Efficacy and safety of carfilzomib in relapsed and/or refractory multiple myeloma: systematic review and meta-analysis of 14 trials (Shah et al. 2018)	SLR	1a			
		A systematic review and meta-analysis of carfilzomib-associated thrombocytopenia as an adverse event in patients with multiple myeloma (Smrdel et al. 2024)	SLR	1a			

		Carfilzomib, dexamethasone, and daratumumab versus carfilzomib and dexamethasone for patients with relapsed or refractory multiple myeloma (CANDOR): results from a randomised, multicentre, open-label, phase 3 study (Dimopoulos et al. 2020)	RCT	1b			
		Isatuximab, carfilzomib, and dexamethasone in relapsed multiple myeloma (IKEMA): a multicentre, open-label, randomised phase 3 trial (Moreau et al. 2021)	RCT	1b			
38	In patients who are anti-CD38 refractory, treatment options include KRd, KPd, and PVd.	NCCN Clinical Practice Guidelines in Oncology (Multiple Myeloma) (NCCN 2025)	CPG	1a	High	82%	NA
		Pomalidomide, bortezomib, and dexamethasone for patients with relapsed or refractory multiple myeloma previously treated with lenalidomide (OPTIMISMM): A randomised, open-label, phase 3 trial (Richardson et al. 2019)	RCT	1b			
		Carfilzomib, lenalidomide, and dexamethasone for relapsed multiple myeloma (Stewart et al. 2015)	RCT	1b			

		An open-label phase 2 study treating patients with first or second relapse of multiple myeloma with carfilzomib, pomalidomide, and dexamethasone (KPd): SELECT study (Perrot et al. 2024)	RCT	1b		
39	Patients with disease refractory to the novel agent in a doublet regimen should be considered for an alternative triplet therapy that excludes the drug they are no longer responding to.	NCCN Clinical Practice Guidelines in Oncology (Multiple Myeloma) (NCCN 2025)	CPG	1a	High	100%	NA
40	For second or subsequent relapses, consider drug classes not used during the first relapse. Regimens containing at least two agents to which the patient is not refractory from the first-relapse options should be prioritised. Clinical trial enrolment is encouraged when feasible. Treatment options include Isa-Pd, DKd, DPd, Isa-Kd, Elo-Pd, and KPd. If daratumumab,	Multiple myeloma: 2024 update on diagnosis, risk stratification, and management (Rajkumar 2024)<br><br>Treatment of relapsed and refractory multiple myeloma: Recommendations from the IMWG (Moreau et al. 2021)<br><br>Recent advances in the management of multiple myeloma: clinical impact based on resource-stratification. Consensus statement of the Asian Myeloma Network at the 16th	IMWG guidelines<br><br>IMWG guidelines<br><br>AMN guidelines	1a<br><br>1a<br><br>1a	High	82%	NA

	carfilzomib, or elotuzumab is unavailable, PCd or Pd may be used. Bendamustine, thalidomide, cyclophosphamide or melphalan-containing regimens can be used for transplant-ineligible patients in resource-constrained settings (Grade of evidence: moderate)	International Myeloma Workshop (Tan et al. 2018)					
		Efficacy of thalidomide-based therapy following lenalidomide plus dexamethasone in patients with relapsed/refractory multiple myeloma (Kukreti et al. 2013)	Retrospective study	2b			
41	In patients who are anti-CD38 refractory and have received two prior lines of therapy, including lenalidomide and a PI, the treatment option is EPd. IxPd is recommended for those who have received two prior lines of therapy containing an IMiD and a PI, and with disease progression on/within 60 days of completion of the last therapy.	NCCN Clinical Practice Guidelines in Oncology (Multiple Myeloma) (NCCN 2025)	CPG	1a	High	82%	NA
		Elotuzumab plus pomalidomide and dexamethasone for multiple myeloma (Dimopoulos et al. 2018)	RCT	1b			
		Multiple myeloma refractory to lenalidomide: A systematic literature review of trials and real-world evidence (Hartley-Brown et al. 2024)	SLR	1a			
42	In enhanced resource settings, CAR T-cell therapy can be an option	NCCN Clinical Practice Guidelines in Oncology (Multiple Myeloma) (NCCN 2025)	CPG	1a	High	73%	91%

	for patients who are refractory after two prior lines of therapy that included an IMiD, an anti-CD38 mAb, and a PI.	Treatment of multiple myeloma in patients refractory to daratumumab/anti-CD38 mAbs: A systematic review (Tan et al. 2025)	SLR	1a			
43	In patients with MM, SC daratumumab (1800 mg) demonstrates a comparable safety profile to IV daratumumab, with shorter median administration time and reduced IRR rates. SC daratumumab is a viable and preferable alternative to IV daratumumab in eligible Asian patients. Also, the combination of daratumumab, thalidomide, and dexamethasone is effective in RRMM. The use of thalidomide and dexamethasone provides a cost-effective option that may improve access to daratumumab-based triplets.	Subcutaneous daratumumab in Asian patients with heavily pretreated multiple myeloma: Subgroup analyses of the noninferiority, phase 3 COLUMBA study (Iida et al. 2021)	RCT	1b	High	82%	NA
		A phase 2 study of daratumumab in combination with thalidomide and dexamethasone in relapsed and/or refractory myeloma: A report from the Asian Myeloma Network (Jen et al. 2023)	RCT	1b			
44	For RRMM patients, perform a thorough evaluation for	NCCN Clinical Practice Guidelines in Oncology (Multiple Myeloma) (NCCN 2025)	CPG	1a	High	91%	NA

	transformation to aggressive disease such as plasma cell leukaemia or extramedullary disease, and consider repeat FISH to assess for t(11;14). For aggressive MM, regimens such as DCEP, DT-PACE, or VTD-PACE may be used. In cases with t(11;14), venetoclax/dexamethasone ± daratumumab or a PI may be considered.	Recent advances in the management of multiple myeloma: clinical impact based on resource-stratification. Consensus statement of the Asian Myeloma Network at the 16th International Myeloma Workshop (Tan et al. 2018)	AMN guidelines	1a			
45	In enhanced-resource settings, approved CAR T-cell therapy is recommended for patients who have relapsed after at least three prior lines of therapy.	NCCN Clinical Practice Guidelines in Oncology (Multiple Myeloma) (NCCN 2025)<br><br>Multiple myeloma: 2024 update on diagnosis, risk stratification, and management (Rajkumar 2024)<br><br>Treatment of multiple myeloma in patients refractory to daratumumab/anti-CD38 mAbs: A systematic review (Tan et al. 2025)	CPG<br><br>IMWG guidelines<br><br>SLR	1a<br><br>1a<br><br>1a	High	55%	91%
46	In limited-resource settings, bendamustine,	Recent advances in the management of multiple	AMN guidelines	1a	Moderate	91%	NA

	thalidomide, cyclophosphamide or melphalan-containing regimens can be used.	myeloma: clinical impact based on resource-stratification. Consensus statement of the Asian Myeloma Network at the 16th International Myeloma Workshop (Tan et al. 2018)					
		Bendamustine-based regimens as salvage therapy in refractory/relapsed multiple myeloma patients: A retrospective real-life analysis by the Polish Myeloma Group (Grzasko et al. 2021)	Retrospective study	2b			
47	In enhanced-resource settings, bispecific antibodies are recommended for patients who have received at least four prior therapies that included an anti-CD38 mAb, a PI, and an IMiD. When feasible, participation in a clinical trial is recommended. Available options in this setting include elranatamab-bcmm,	NCCN Clinical Practice Guidelines in Oncology (Multiple Myeloma) (NCCN 2025)	CPG	1a	High	73%	100%
		Treatment of multiple myeloma in patients refractory to daratumumab/anti-CD38 mAbs: A systematic review (Tan et al. 2025)	SLR	1a			

	talquetamab-tgvs, and teclistamab-cqyv.						
48	Selinexor in combination with dexamethasone may be considered for patients who have received at least four prior therapies and whose disease is refractory to at least two PIs, two IMiDs, and an anti-CD38 mAb.	NCCN Clinical Practice Guidelines in Oncology (Multiple Myeloma) (NCCN 2025)	CPG	1a	High	82%	NA
		Oral selinexor-dexamethasone for triple-class refractory multiple myeloma (Chari et al. 2019)	RCT	1b			
		Once-per-week selinexor, bortezomib, and dexamethasone versus twice-per-week bortezomib and dexamethasone in patients with multiple myeloma (BOSTON): a randomised, open-label, phase 3 trial (Grosicki et al. 2020)	RCT	1b			
49	For patients who have received at least four prior therapies, including an anti-CD38 mAb, a PI, and an IMiD, belantamab mafodotin-blmf may be considered if available. Patients may be treated with more than one BCMA-targeted therapy for their disease. Although the optimal sequencing of BCMA-	NCCN Clinical Practice Guidelines in Oncology (Multiple Myeloma) (NCCN 2025)	CPG	1a	High	73%	91%
		Treatment of multiple myeloma in patients refractory to daratumumab/anti-CD38 mAbs: A systematic review (Tan et al. 2025)	SLR	1a			
		Belantamab Mafodotin, Bortezomib, and Dexamethasone	RCT	1b			

	directed therapies is not yet established, emerging data suggest that immediate sequential use after relapse may result in lower response rates.	for Multiple Myeloma (Hungria et al. 2024)					
		Ocular adverse events associated with antibody-drug conjugates in oncology: A pharmacovigilance study based on the FDA adverse event reporting system (FAERS) (Mao et al. 2024)	Retrospective studies	2b			
50	During periods of increased infectious risk, antibacterial prophylaxis is recommended. Prophylactic antiviral treatment (Acyclovir) is also recommended for patients undergoing a regimen that includes a PI or CD38-targeted therapy.	NCCN Clinical Practice Guidelines in Oncology (Multiple Myeloma) (NCCN 2025)	CPG	1a	High	91%	NA
		Consensus guidelines and recommendations for infection prevention in multiple myeloma: A report from the IMWG (Raje et al. 2022)	IMWG guidelines	1a			
		Expert consensus on the incorporation of anti-CD38 mAb therapy into the mana gement of newly diagnosed multiple myel oma (Lonial et al. 2023)	Consensus guidelines	5			
51	Patients with recurrent infections should undergo an evaluation of their immune status, including a detailed infection history and	Consensus guidelines and recommendations for infection prevention in multiple myeloma: A report from the IMWG (Raje et al. 2022)	IMWG guidelines	1a	High	91%	NA

	laboratory testing of immune parameters such as immunoglobulin levels, to determine eligibility for early IVIG therapy. Although the optimal IVIG dosing schedule is not clearly established, the treatment goal should be to prevent further infections.	Expert consensus on the incorporation of anti CD38 mAb therapy into the mana gement of newly diagnosed multiple myel oma (Lonial et al. 2023)	Consensus guidelines	5			
52	Hypogammaglobulinaemia is more common in patients treated with daratumumab. IVIG replacement should be considered based on individual risk, with IgG levels monitored to guide use. Primary IVIG supplementation has also been shown to reduce high-grade infections in patients receiving teclistamab. Further studies are needed to better identify patients at the highest infection risk and optimise IVIG use.	Consensus guidelines and recommendations for infection prevention in multiple myeloma: A report from the IMWG (Raje et al. 2022)	IMWG guidelines	1a	High	82%	NA
		Expert consensus on the incorporation of anti-CD38 mAb therapy into the management of newly diagnosed multiple myel oma (Lonial et al. 2023)	Consensus guidelines	5			
		Effects of IVIG supplementation (IVIG) on infections in recipients of teclistamab therapy for multiple myeloma (MM): A multi-institutional study (Cheruvalath et al. 2024)	Retrospective study	2b			

		Hypogammaglobulinaemia and its implications in patients treated with daratumumab: A single institution experience (Paul et al. 2019)	Retrospective study	2b			
		Effect of IVIG on infections in multiple myeloma (MM) patients receiving daratumumab (Lancman et al. 2020)	Retrospective study	2b			
53	Considering the enhanced safety profile of subcutaneously administered daratumumab, prolonged use of corticosteroids solely to prevent IRR is not advised.	Subcutaneous versus intravenous daratumumab in patients with relapsed or refractory multiple myeloma (COLUMBA): a multicentre, open-label, noninferiority, randomised, phase 3 trial (Mateos et al. 2020)	RCT	1b	High	100%	NA
		Subcutaneous daratumumab with rapid corticosteroid tapering in relapsed or refractory multiple myeloma patients: part 3 update of the open-label, multicentre, Phase 1b Pavo study (Nahi et al. 2021)	Clinical trial	2b			
		Expert consensus on the incorporation of anti-CD38 mAb therapy into the management of newly diagnosed multiple myel oma (Lonial et al. 2023)	Consensus guidelines	5			

54	Vaccinations play a crucial role in an effective infection prevention strategy for all patients. HCPs should consult the most up-to-date recommendations on seasonal vaccines and other vaccines relevant to adult patients. The timing of vaccinations should be considered based on the patient's MM burden or disease response, as well as the time since receiving anti-myeloma treatments, especially high-dose chemotherapy or ASCT.	Consensus guidelines and recommendations for infection prevention in multiple myeloma: A report from the IMWG (Raje et al. 2022)<br><br>Expert consensus on the incorporation of anti-CD38 mAb therapy into the management of newly diagnosed multiple myeloma (Lonial et al. 2023)	IMWG guidelines<br><br>Consensus guidelines	1a<br><br>5	High	100%	NA
55	Patients with high-risk SMM may benefit from early intervention, with single-agent lenalidomide as an option for carefully selected cases. Those with rising biomarkers or evolving risk features need close monitoring as low-risk disease can progress to	NCCN Clinical Practice Guidelines in Oncology (Multiple Myeloma) (NCCN 2025)<br><br>Daratumumab or active monitoring for high-risk SMM (Dimopoulos et al. 2024)	CPG<br><br>RCT	1a<br><br>1b	High	82%	100%

	high-risk over time, necessitating adjusted follow-up and management strategies. Daratumumab monotherapy can also be considered as an alternative.						
56	All patients receiving initial therapy for MM should receive bone-targeting agents, such as bisphosphonates, for up to 2 years. Dosing frequency (monthly vs. every 3 months) should be individualised based on patient factors, treatment response, and the chosen agent. Extensions beyond 2 years should be guided by clinical judgment. Denosumab is an alternative option to bisphosphonates.	NCCN Clinical Practice Guidelines in Oncology (Multiple Myeloma) (NCCN 2025)<br><br>Denosumab versus zoledronic acid in bone disease treatment of newly diagnosed multiple myeloma: An international, double-blind, randomised controlled phase 3 study-Asian subgroup analysis (Huang et al. 2020)	CPG<br><br>RCT	1a<br><br>1b	High	100%	NA
57	Radiation therapy may be considered for patients with pain that does not respond to systemic treatment or in cases of bone lesions causing	Radiation therapy for solitary plasmacytoma and multiple myeloma: Guidelines from the International Lymphoma Radiation Oncology Group (Tsang et al. 2018)	ILROG guidelines	1a	Moderate	100%	NA

	spinal cord compression. If the compression is due to retropulsed bone, surgery should be prioritised over radiation. Extensive use of radiotherapy, especially early in the disease for pain relief alone, should be avoided to preserve bone marrow function.	Management of myeloma manifestations and complications: The cornerstone of supportive care: Recommendation of the Canadian Myeloma Research Group (formerly Myeloma Canada Research Network) Consensus Guideline Consortium (LeBlanc et al. 2022)	Consensus guidelines	5			
58	Orthopaedic consultation should be sought without delay for fractures, impending fractures, or instability in weight-bearing bones to consider surgical stabilisation.	Radiation therapy for solitary plasmacytoma and multiple myeloma: Guidelines From the International Lymphoma Radiation Oncology Group (Tsang et al. 2018)	ILROG guidelines	1a	Moderate	100%	NA
	Postoperative radiation is rarely required in these cases, as systemic therapy typically provides adequate disease control.	Management of myeloma manifestations and complications: The cornerstone of supportive care: Recommendation of the Canadian Myeloma Research Group (formerly Myeloma Canada Research Network) Consensus Guideline Consortium (LeBlanc et al. 2022)	Consensus guidelines	5			

AMN = Asian Myeloma Network; APAC = Asia-pacific; ASCT = autologous stem cell transplantation; ASTCT = American Society for Transplantation and Cellular Therapy; BCMA = B-cell maturation antigen; BMPCs = bone marrow plasma cells; CAR T = chimeric antigen receptor t cell; CD38 = cluster of differentiation 38; CPG = Clinical Practice Guideline; CR = complete response; CSS = conventional skeletal

survey; CXCR4 = c-x-c chemokine receptor type 4; CyBorD = cyclophosphamide, bortezomib, and dexamethasone; DCEP = dexamethasone, cyclophosphamide, etoposide, and cisplatin; DCVRd = daratumumab, cyclophosphamide, bortezomib, lenalidomide, and dexamethasone; DKd = daratumumab, carfilzomib, and dexamethasone; DKRd = daratumumab, carfilzomib, lenalidomide, and dexamethasone; DpR = depth of response; DPd = daratumumab, pomalidomide, and dexamethasone; DRd = daratumumab, lenalidomide, and dexamethasone; DT-PACE = dexamethasone, thalidomide, cisplatin, doxorubicin, cyclophosphamide, and etoposide; DVd = daratumumab, bortezomib, and dexamethasone; DVRd = daratumumab, bortezomib, lenalidomide, and dexamethasone; DVTd = daratumumab, bortezomib, thalidomide, dexamethasone; EHA = European Haematology Association; Elo-Pd = elotuzumab, pomalidomide, and dexamethasone; EPd = elotuzumab, pomalidomide, and dexamethasone; ESMO = European Society for Medical Oncology; FAERS = Food and Drug Administration Adverse Event Reporting System; FISH = fluorescence in situ hybridisation; FLC = free light chain; FLCr = free light chain ratio; G-CSF = granulocyte colony-stimulating factor; HCT = hematopoietic cell transplantation; HCPs = health care professionals; HSC = hematopoietic stem cell; HSCT = hematopoietic stem cell transplantation; IgG = immunoglobulin g; IgH = immunoglobulin heavy chain; ILROG = International Lymphoma Radiation Oncology Group; IMiD = immunomodulatory drug; IMWG = International Myeloma Working Group; IRR = infusion-related reaction; Isa-Kd = isatuximab, carfilzomib, and dexamethasone; Isa-KRd = isatuximab, carfilzomib, lenalidomide, and dexamethasone; Isa-Pd = isatuximab, pomalidomide, and dexamethasone; Isa-VRd = isatuximab, bortezomib, lenalidomide, and dexamethasone; ISS = International Staging System; IV = intravenous; IVIG = intravenous immunoglobulin; IxPd = ixazomib, pomalidomide, and dexamethasone; KRd = carfilzomib, lenalidomide, and dexamethasone; KPd = carfilzomib, pomalidomide, and dexamethasone; LDH = lactate dehydrogenase; mAb = monoclonal antibody; MASS = Mayo Additive Staging System; MDEs = myeloma-defining events; mSMART = Mayo Stratification for Myeloma and Risk-Adapted Therapy; MM = multiple myeloma; MPT = melphalan, prednisone, and thalidomide; MR = minimal response; MRD = minimal residual disease; MRDR = myeloma and related diseases registry; MRI = magnetic resonance imaging; NA = not applicable; NCCN = National Comprehensive Cancer Network; NDMM = newly diagnosed multiple myeloma; NGF = next-generation flow; NGS = next-generation sequencing; OS = overall survival; PCd = pomalidomide, cyclophosphamide, and dexamethasone; Pd = pomalidomide; PET/CT = positron emission tomography/computed tomography; PFS = progression-free survival; PI = proteasome inhibitor; PR = partial response; PVd = pomalidomide, bortezomib, and dexamethasone; R-ISS = Revised International Staging System; RCT = randomised controlled trial; Rd = lenalidomide and dexamethasone; RR = response rate; RRMM = relapsed or refractory multiple myeloma; sCR = stringent complete response; SC = subcutaneous; SD = stable disease; sFLC = serum free light chain; SIFE = serum immunofixation electrophoresis; SLiM = sixty percent, light chain, magnetic resonance imaging; SLR = systematic literature review; SMM = smouldering multiple myeloma; SPEP = serum protein electrophoresis; TIE = transplant-ineligible; TP53 = tumour protein p53; UK = United Kingdom; VCd = bortezomib, cyclophosphamide, and dexamethasone; VCD-VTD = bortezomib, cyclophosphamide, dexamethasone followed by bortezomib, thalidomide, and dexamethasone; Vd = bortezomib and dexamethasone; VES-13 = Vulnerable Elders Survey-13; VGPR = very good partial response; VMP = bortezomib, melphalan, and prednisone; VRd = bortezomib, lenalidomide, and dexamethasone; VTd = bortezomib, thalidomide, and dexamethasone; VTD-PACE = bortezomib, thalidomide, dexamethasone, cisplatin, doxorubicin, cyclophosphamide, and etoposide; WB-CT = whole-body computed tomography.
